# Pathology-tailored nanotherapy via Galectin-3-targeted and triple-responsive nanoparticles enables multimodal therapy against aortic dissection

**DOI:** 10.1186/s12951-025-04012-7

**Published:** 2026-01-13

**Authors:** Chi Lin, Min-Lang Tsai, Hsin-Yi Chao, Tsai-Mu Cheng, Chun-Ming Shih, Alexander T. H. Wu, Chia-Hsiung Cheng, Chen Yuan Hsiao, Hsin-Ying Lu, Chun-Che Shih, Fwu-Long Mi

**Affiliations:** 1 Graduate Institute of Nanomedicine and Medical Engineering, College of Biomedical Engineering, Taipei Medical University, Taipei, 11031 Taiwan; 2 Department of Biochemistry and Molecular Cell Biology, School of Medicine, College of Medicine, Taipei Medical University, Taipei, 11031 Taiwan; 3 Department of Food Science, National Taiwan Ocean University, Keelung, 20224 Taiwan, ROC; 4 The PhD Program for Translational Medicine, College of Medical Science and Technology, Taipei Medical University, Taipei, 11031 Taiwan; 5 Division of Cardiology and Cardiovascular Research Center, Taipei Medical University Hospital, Taipei, 11031 Taiwan; 6 Graduate Institute of Medical Sciences, College of Medicine, Taipei Medical University, Taipei, 11031 Taiwan; 7 Present Address: Taipei Heart Institute, Taipei Medical University, Taipei, 11031 Taiwan; 8 Department of Surgery, School of Medicine, College of Medicine, Taipei Medical University, Taipei, 11031 Taiwan; 9 Division of Cardiovascular Surgery, Department of Surgery, Wan Fang Hospital, Taipei Medical University, Taipei, 11696 Taiwan

**Keywords:** Galectin-3 targeting, Modified citrus pectin, Self-assembly, Pathology-specific nanotherapy, Aortic dissection

## Abstract

Aortic dissection (AD) is a life‑threatening vascular disorder with high mortality and no effective pharmacological treatment. Addressing its multicellular and dynamic pathology requires strategies that precisely modulate inflammatory and degenerative processes, yet existing targeted delivery approaches lack the spatiotemporal and cellular precision required. Here, we establish Galectin‑3 (Gal‑3) as a therapeutic delivery target for cardiovascular nanomedicine and introduce a modular, pathology‑tailored nanoplatform synergizing nitric oxide (NO) therapy with multi‑pathway intervention. Gal‑3 is persistently expressed on inflamed endothelial cells, macrophages, and smooth muscle cells during AD progression, providing a tractable target for lesion‑specific engagement. The nanoparticles, created from a previously unexplored integration of a Gal‑3‑binding polysaccharide, a nitric oxide–generating peptide, and a hydrophobic drug carrier, uniquely combine triple responsiveness to pH, protease, and oxidative stress with on‑demand NO release and controlled resveratrol co‑delivery. In vitro, they enhanced uptake across pathological cell types and attenuated inflammatory and degenerative phenotypes. In vivo, they achieved early lesion targeting, > 20‑fold aortic accumulation, and marked reductions in AD incidence, vascular degeneration, and mortality. This work establishes Gal‑3‑targeted nanotherapy as a broadly applicable paradigm for pathology‑adaptive intervention in AD and one that may be adapted for broader cardiovascular applications.

**Graphical Abstract:**

**Figure:**
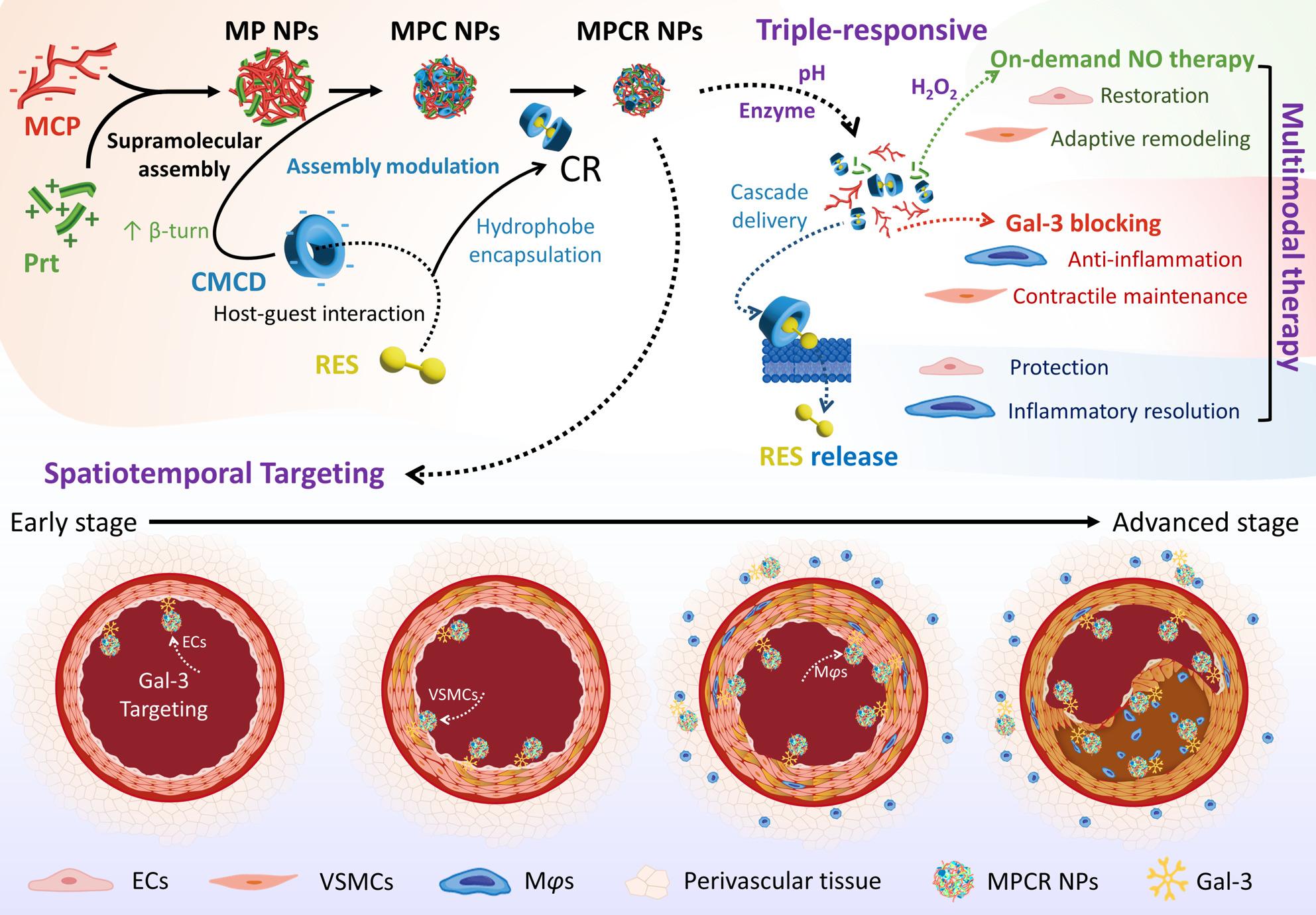
Schematic 1. Schematic illustration of a triple-responsive nanoplatform (MPCR NPs) that enables pathology-tailored targeting via Gal-3 recognition and achieves multimodal therapy for AD. MPCR NPs are constructed from MCP, Prt, and CR through supramolecular co-assembly primarily driven by electrostatic and hydrogen-bonding interactions between MCP and Prt. CMCD modulates the assembly by increasing Prt β-turn conformation, thereby reducing particle size. Upon exposure to pathological stimuli (acidic pH, protease, and H2O2), MPCR NPs undergo cascade release of CR and subsequent RES liberation. The system enables spatiotemporal targeting across ECs, VSMCs, and Mφs during AD progression. Cell-specific therapeutic effects include: (1) on-demand NO therapy for EC restoration and VSMC adaptive remodeling; (2) Gal-3 blocking for inflammation attenuation and contractile phenotype maintenance; and (3) RES-mediated protection of ECs and Mφs, leading to oxidative stress reduction and inflammatory resolution. Collectively, MPCR NPs provide a pathology-adaptive nanotherapeutic strategy with spatiotemporally precise delivery and multi-pathway intervention.

**Supplementary Information:**

The online version contains supplementary material available at 10.1186/s12951-025-04012-7.

## Introduction

Aortic dissection (AD) is a catastrophic vascular condition characterized by the separation of aortic wall layers, often resulting in limited therapeutic options and high mortality [1, 2]. It is classified into type A, requiring immediate surgery, and type B, which is typically managed with antihypertensive therapy unless complications arise [1]. While surgical and endovascular interventions stabilize acute cases, they do not address the underlying vascular degeneration, resulting in high recurrence rates and reduced survival [1, 3]. Current pharmacological management, limited to blood pressure and lipid control, offers only marginal benefits and fails to directly target disease progression [4, 5]. Therefore, developing novel therapeutic strategies is critical to overcoming these limitations and improving long-term outcomes.

To address these challenges, targeted drug delivery offers a promising approach to selectively act on pathological sites within the aortic wall. By addressing key drivers of AD progression, such an approach may provide superior disease modulation beyond conventional systemic therapies. Recent advances in targeted drug delivery systems have explored various strategies, including cyclic arginyl-glycyl-aspartic acid (cRGD) peptide-modified nanoparticles (NPs), which target the αVβ3 integrin overexpressed at thoracic AD (TAD) sites [6], and activated neutrophil membrane-coated NPs (NMCs), which leverage homotypic attraction to accumulate at sites of AD or aneurysm [7]. NMCs effectively localize to inflamed sites by leveraging neutrophil-driven early-stage AD, making them well-suited for acute-phase drug delivery. However, the transient targeting efficacy of NMCs [8, 9], along with their complex fabrication, batch variability, instability, and potential immunogenicity of cell membrane-coated NPs, limits scalability and hinders their clinical translation [10, 11]. Similarly, cRGD peptide-modified NPs face limitations, as the αVβ3 integrin is primarily linked to neovascularization and cellular migration rather than early inflammatory processes, restricting their effectiveness in targeting early-stage AD [9].

Galectin-3 (Gal-3) has emerged as a promising target for drug delivery due to its sustained involvement across all stages of AD. Unlike transient inflammatory markers or homotypic attraction, which primarily facilitate acute-phase targeting [8, 9], Gal-3 remains consistently expressed, enabling both early intervention and long-term disease modulation [12–15]. In early-stage AD, Gal-3 is upregulated in endothelial cells (ECs), promoting endothelial dysfunction and the endothelial-to-mesenchymal transition, thereby fostering a pro-inflammatory microenvironment, as observed in vascular disorders linked to AD, such as arterial hypertension and vascular inflammation [14–16]. As AD progresses, macrophage (Mφ)-driven inflammation further amplifies Gal-3 expression, enhancing chemokine production and monocyte recruitment, consistent with findings in AD models and related vascular disorders, including atherosclerosis and aortic aneurysms [12, 13, 15, 17]. In later stages, Gal-3 accumulates in vascular smooth muscle cells (VSMCs), driving extracellular matrix (ECM) remodeling, phenotypic transition, and fibrosis, aligning with observations from AD and arterial remodeling studies [13, 15, 18–22]. This sustained expression across pathological cell types positions Gal-3 as a strategic entry point for pathology-tailored therapeutic delivery in AD. Herein, we propose a Gal-3-mediated strategy designed to selectively engage pathological cells and disease loci throughout AD progression, offering a paradigm shift in AD-targeted drug delivery by enabling both a spatially and temporally sustained therapeutic intervention.

Gal-3-targeted nanosystems demonstrated successful targeted delivery and therapeutic efficacy in cancer, neural, and fibrosis therapies [23–28]. One promising approach for Gal-3-targeted therapy is the use of modified citrus pectin (MCP), a well-established Gal-3 inhibitor with high binding affinity [29, 30], making it an ideal candidate for site-specific drug delivery [25, 26, 28]. By leveraging the strong affinity of MCP for Gal-3, novel drug delivery systems can achieve not only precise early-stage targeting and also sustained modulation of disease progression. Furthermore, in precursor conditions of AD, including atherosclerosis, aortic aneurysm, and hypertension-induced vascular remodeling, MCP inhibits Gal-3-mediated endothelial dysfunction, Mφ-driven inflammation, and ECM degradation, highlighting its potential to stabilize the aortic wall, prevent dissection, and mitigate long-term vascular remodeling [31].

A pathology-tailored drug delivery system enables previously unstable or systemically limited therapeutic molecules to overcome inherent challenges and emerge as viable candidates for AD treatment. Among potential pharmacological strategies, nitric oxide (NO) therapies hold considerable promise for vascular modulation in AD. However, their clinical application is hindered by NO’s short biological half-life, rapid systemic diffusion, and susceptibility to degradation in the bloodstream, necessitating a controlled and localized release system to ensure sustained efficacy while minimizing off-target effects [32, 33].

An NO self-generating delivery system offers a strategy to maintain localized NO availability while overcoming limitations of exogenous NO donors. These platforms leverage microenvironmental cues, such as oxidative stress, inflammation, and enzymatic activity, to achieve spatiotemporally controlled NO release, thereby regulating vascular tone, modulating inflammation, and orchestrating ECM remodeling by targeting key vascular cells such as VSMCs, Mφs, and ECs [32–35]. To adapt this strategy to the context of AD, localized NO delivery can be effectively integrated into the previously proposed Gal-3-targeted, pathology-tailored drug delivery system, enabling sustained and selective NO release at pathological sites. Specifically, in ECs, localized NO release improves endothelial function and mitigates oxidative stress [33, 34], thereby reducing dysfunction that contributes to AD onset. In Mφs, controlled NO exposure modulates inflammatory responses, balancing pro-inflammatory and reparative phenotypes while limiting ECM degradation [35]. In VSMCs, NO delivery inhibits pathological phenotypic switching, suppresses proliferation, and prevents apoptosis, ultimately enhancing arterial wall stability [32, 36]. To date, no studies have directly investigated localized NO delivery in AD. However, clinical observations of inhaled NO showed modest therapeutic benefits [37, 38], indicating a potential role for NO in AD management. This highlights the rationale for developing targeted and sustained NO delivery strategies tailored to AD’s pathology to address the current lack of effective pharmacological interventions.

Beyond NO regulation, this targeting system can also serve as a versatile carrier for additional therapeutic agents. Resveratrol (RES), a natural polyphenolic compound, has shown therapeutic efficacy in preclinical models of AD. In β-aminopropionitrile (BAPN)-induced AD mice, RES significantly reduced the dissection incidence by enhancing endothelial barrier function and suppressing oxidative stress and inflammation via SIRT1 activation [39, 40]. In Marfan syndrome models, RES further attenuated aortic dilatation by reducing protease activity, alleviating oxidative damage, and promoting VSMC survival [41, 42]. RES also modulates Mφ polarization toward an anti-inflammatory phenotype, thereby limiting ECM degradation and chronic vascular inflammation during AD progression [40]. Despite its promising effects, clinical translation of RES has been severely limited by intrinsic pharmacokinetic drawbacks, such as poor solubility, rapid metabolism, and low bioavailability, which result in subtherapeutic drug levels at pathological sites [43, 44]. As a consequence, clinical trials for cardiovascular applications have often yielded suboptimal or inconsistent outcomes due to insufficient systemic exposure and lack of targeted delivery [45]. Given its demonstrated efficacy across multiple pathological cell types and its pharmacokinetic drawbacks, RES represents a compelling candidate for incorporation into the Gal-3-targeted, pathology-tailored delivery strategy, which is well-suited for achieving cell-selective and sustained drug delivery in AD.

To address the complex therapeutic demands of AD, we designed a Gal-3-targeted, pathology-tailored delivery platform that enables spatially specific and controlled release of therapeutic agents. Given that AD lesions are characterized by elevated oxidative stress and localized acidosis [46, 47], this nanosystem was constructed via electrostatic self-assembly between MCP and protamine (Prt), an NO-donating peptide. Prt imparts the NPs with sensitivity to enzymatic degradation and oxidative stress, particularly hydrogen peroxide, enabling environmentally triggered NO supply [33]. This stimulus-responsive behavior underscores the platform’s therapeutic potential for inflammation-associated vascular disorders such as AD. In addition, carboxymethyl-β-cyclodextrin (CMCD) was strategically utilized to encapsulate RES, thereby improving its aqueous compatibility and enabling pH-responsive controlled release [48, 49].

This modular co-assembly strategy integrated CMCD-RES complexes, MCP, and Prt into a unified nanoplatform, where each component plays distinct therapeutic and functional roles. We hypothesized that a Gal-3-targeted nanoplatform, engineered to co-deliver NO and RES with spatial specificity and pathological cue-responsive release, could modulate AD-relevant pathological processes by selectively targeting key cellular drivers across disease stages (Scheme 1). To our knowledge, there are currently no existing reports that utilize Gal-3 as a targeting strategy for cardiovascular drug delivery or combine MCP with NO-donor materials into a co-assembled nanoplatform. This orchestrated design not only addresses the pharmacokinetic limitations of RES by enhancing therapeutic availability and thereby enabling lesion-selective release, but also establishes a versatile platform for coordinated multi-functional therapy within pathological environments, offering substantial potential to improve both early-stage disease mitigation and long-term vascular stabilization in AD.

**Fig. 1:**
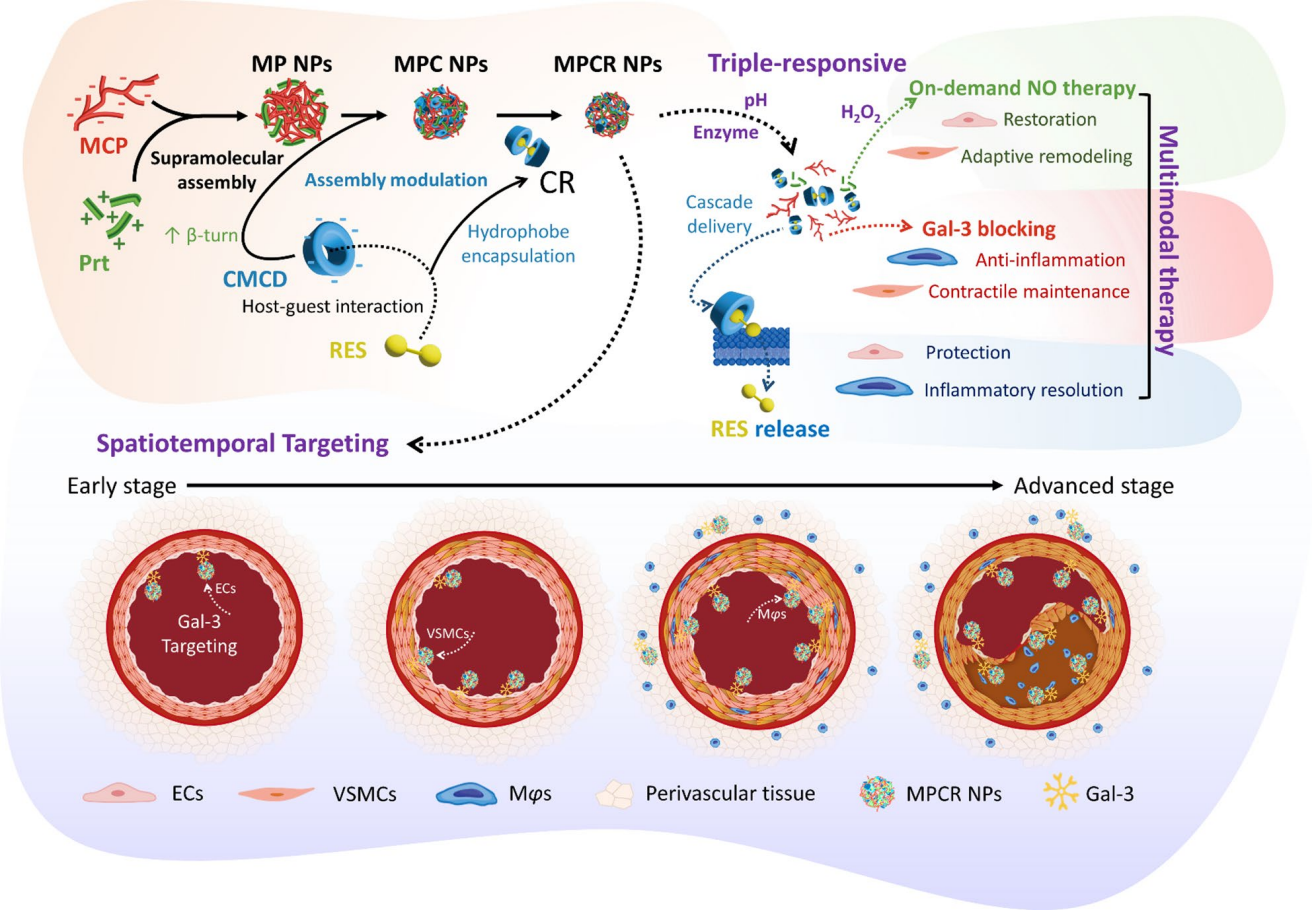
Schematic illustration of a triple-responsive nanoplatform (MPCR NPs) that enables pathology-tailored targeting via Gal-3 recognition and achieves multimodal therapy for AD. MPCR NPs are constructed from MCP, Prt, and CR through supramolecular co-assembly primarily driven by electrostatic and hydrogen-bonding interactions between MCP and Prt. CMCD modulates the assembly by increasing Prt β-turn conformation, thereby reducing particle size. Upon exposure to pathological stimuli (acidic pH, protease, and H_2_O_2_), MPCR NPs undergo cascade release of CR and subsequent RES liberation. The system enables spatiotemporal targeting across ECs, VSMCs, and M*φ*s during AD progression. Cell-specific therapeutic effects include: (1) on-demand NO therapy for EC restoration and VSMC adaptive remodeling; (2) Gal-3 blocking for inflammation attenuation and contractile phenotype maintenance; and (3) RES-mediated protection of ECs and M*φ*s, leading to oxidative stress reduction and inflammatory resolution. Collectively, MPCR NPs provide a pathology-adaptive nanotherapeutic strategy with spatiotemporally precise delivery and multi-pathway intervention.

## Results and discussion

### Modular assembly of MCP-based NPs

**Fig. 2:**
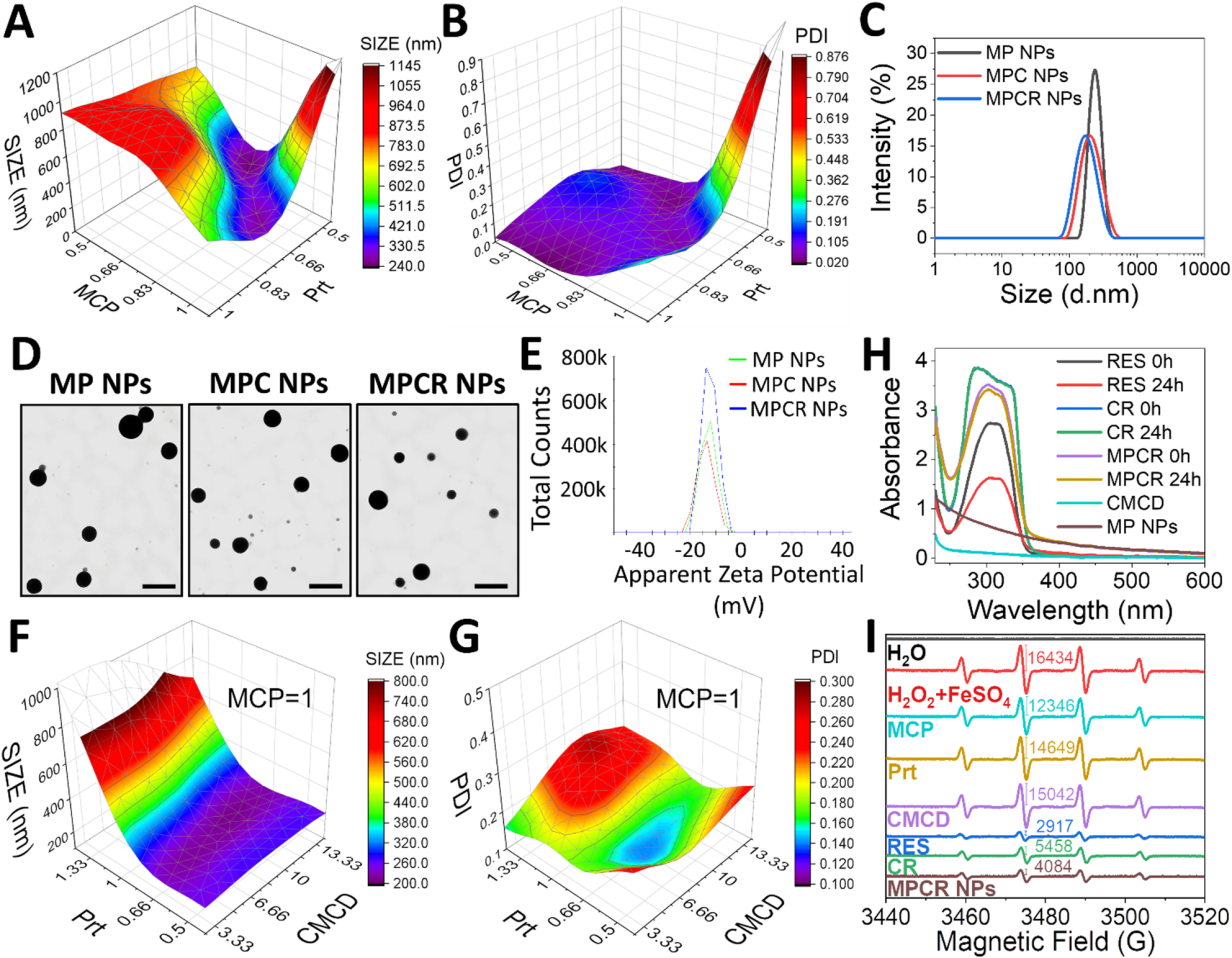
Fabrication and optimization of nanoparticles. Effects of MCPD and Prt ratios on nanoparticle size (**A**) and PDI (**B**). (**C**) TEM images (scale bars: 500 nm). (**D**) DLS size distributions. (**E**) UV–Vis spectra of individual components and MPCR NPs in PBS (pH 7.4, 37 °C) at 0 and 24 h. (**F**, **G**) Effects of Prt and CMCD ratios on the size (**F**) and PDI (**G**) at fixed MCP = 1. (**H**) UV-vis spectrum of samples in PBS. (**I**) EPR spectra showing •OH-scavenging capability under H_2_O_2_/FeSO_4_-induced radical generation, with the RES-equivalent concentration fixed at 16 µM. Original data for (**A**), (**B**), (**F**), and (**G**) are listed in Table S3

**Fig. 3:**
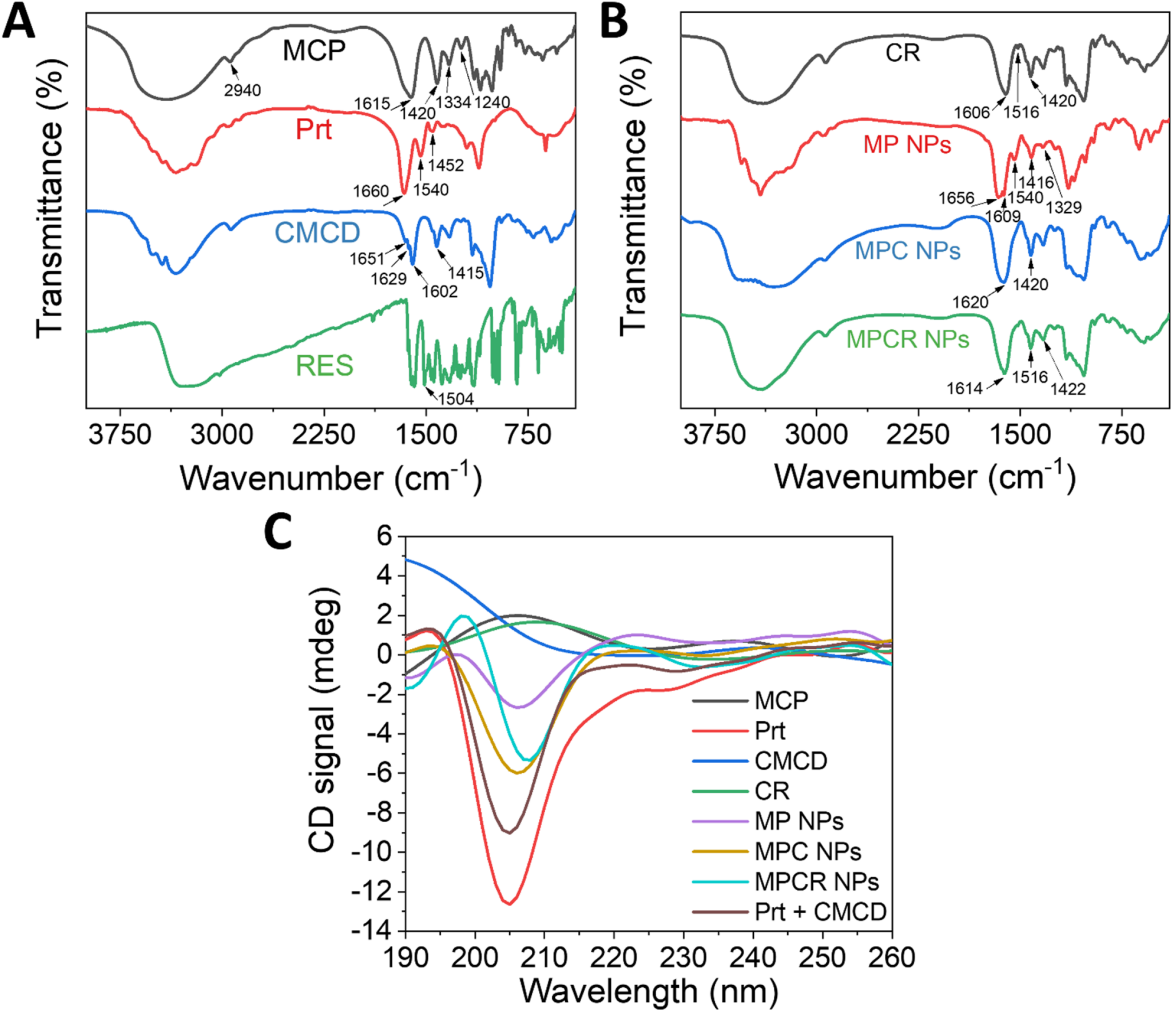
Spectroscopic characterization. FTIR spectra (**A**, **B**) and CD spectra (**C**) of individual components and nanoparticles

To our knowledge, there are no previous reports of pectin-based NPs co-assembled with Prt. In this study, we employed MCP (Mn: 12.6 kDa, 7.5% degree of esterification; Figs. S1, S2), a structurally distinct derivative of natural citrus pectin (CP), characterized by improved aqueous solubility, lower viscosity, reduced molecular weight, and significantly decreased esterification. These physicochemical attributes differentiate MCP from conventional pectin, positioning it as a promising candidate for forming polyelectrolyte-based NPs. Figure 2A demonstrates the co-assembly behavior of MCP and Prt, evaluated by the hydrodynamic particle size. A distinct diagonal valley region, spanning MCP: Prt weight ratios from approximately 0.66:0.83 to 1:0.5, was observed, with the minimal particle size achieved at MCP: Prt = 1:0.66. A complementary analysis using the polydispersity index (PDI) further refined this optimal formulation window, indicating superior NP uniformity within a narrower range ending around MCP: Prt = 1:0.83. Accordingly, the optimal NP formulation, designated MP NPs, was determined at MCP: Prt = 1:0.66, exhibiting a hydrodynamic diameter of 240 nm, a PDI of 0.07 (Fig. 2A, B, Table S1), and a monodispersed particle size distribution (Fig. 3C). TEM images confirmed that the MP NPs were compact, spherical NPs (Fig. 2D). Their moderate negative surface charge (− 12.8 mV, Fig. 2E) indicated MCP dominance on the NP surface.

To broaden the applicability of the MP NP platform and address the challenge of delivering hydrophobic drugs, which comprise most AD therapeutic candidates, we incorporated a cyclodextrin possessing hydrophobic cavities into the existing formulation. Specifically, CMCD, characterized by abundant carboxyl groups, was introduced to enhance NP assembly via robust electrostatic interactions with the strongly cationic Prt, thereby improving the drug-loading capacity and enabling pH-responsive release. As shown in Fig. 2F, although the Prt content exhibited a stronger influence on the particle size, CMCD actively contributed to NP assembly and structural regulation. At a fixed MCP: Prt ratio of 1:0.5, an increasing CMCD content led to a modest size increase, indicating its involvement in particle organization even under low Prt conditions. More importantly, across all tested conditions, incorporating CMCD consistently resulted in significantly smaller particles compared to the optimized MCP: Prt = 1:0.66 formulation, underscoring its dual role as a structural compactor and size modulator. These results highlight the critical role of CMCD not only as a hydrophobic drug carrier but also as an active modulator of NP physicochemical properties. Considering the particle size, PDI, and CMCD content, the optimal formulation was identified at a weight ratio of MCP: Prt: CMCD = 1:0.67:6.67, designated MPC NPs. While the addition of CMCD slightly increased the PDI to 0.13, the hydrodynamic diameter significantly decreased to 215 nm (Fig. 2F, G, Table S1). MPC NPs also retained a monodispersed size distribution (Fig. 2C), exhibited a compact spherical morphology (Fig. 2D), and showed a moderately negative surface charge of − 13.6 mV (Fig. 2E).

To assess the capacity of MPC NPs for hydrophobic drug delivery, RES was incorporated via host-guest complexation with CMCD to form an inclusion complex (CR). ^1^H NMR spectroscopy (Fig. S3) confirmed RES inclusion within the CMCD cavity, showing characteristic chemical shift changes in CMCD protons. Notably, protons located inside the cavity (H3, H5) and near the narrow rim (H6) exhibited upfield shifts, while those near the wider rim (H2, H4) remained largely unchanged. This pattern is consistent with the characteristic inclusion behavior of β-cyclodextrin, in which cavity protons exhibit chemical shift changes due to the anisotropic ring current effect induced by aromatic guest molecules [50]. These results confirmed the successful encapsulation of RES into the CMCD cavity. In the FTIR spectrum of CMCD (Fig. 3A), a broad band appeared in the 1600–1660 cm^− 1^ region, with discernible peaks at 1651 and 1629 cm^− 1^ within the shoulder region, suggesting overlapping vibrational modes. This band was primarily attributed to the asymmetric stretching of carboxylate groups, possibly overlapping with C = O stretching of residual carboxylic acids. A distinct peak at 1415 cm^− 1^ corresponded to symmetric -COO^−^ stretching, confirming the presence of carboxymethyl functionalities. Upon CR complex formation, the original peaks at 1651, 1629, and 1602 cm^− 1^ merged into a single broad band centered at 1606 cm^− 1^, indicating environment-sensitive shifts in -COO^−^ vibrations due to host–guest interactions [51]. The symmetric -COO^−^ band also shifted from 1420 to 1415 cm^− 1^, further supporting changes in the molecular environment and hydrogen bonding network [51]. In addition, the aromatic C = C stretching vibration of RES shifted from 1509 to 1516 cm^− 1^ upon complexation, likely due to suppression of π–π stacking and conformational changes induced by inclusion. These spectral changes collectively confirmed the formation of the CR inclusion complex. The RES loading capacity was approximately 38 µg/mg CMCD, corresponding to a 66-fold enhancement in aqueous solubility (Table S1). Furthermore, CR effectively reduced intracellular ROS, oxidative stress, and inflammatory markers (SOD2, COX2, ICAM-1, VCAM-1), while enhancing cell viability in inflamed ECs, suggesting preservation of RES bioactivity and its release upon cellular contact (Fig. S4).

Upon replacing free CMCD with its drug-loaded counterpart, CR, during co-assembly with MCP and Prt, the resulting MPCR NPs exhibited more-pronounced changes in NP characteristics compared to the original MPC NPs. Specifically, the particle size further decreased to 192 nm, while the PDI slightly increased to 0.15 (Table S1). This effect may be attributed to the larger molecular structure of CR, in which RES is dually included via both A- and B-rings, thereby enhancing the structural influence of CMCD on NP assembly. The encapsulation efficiency of RES in the CR was 95.6%, corresponding to a loading capacity of 30.2 µg RES per mg MPCR NPs. Furthermore, MPCR NPs significantly reduced RES auto-oxidation (Fig. 2H), while preserving its free-radical scavenging activity (Fig. 2I). In Fig. 2H, CR exhibited strong protective effects against RES oxidation, likely due to efficient inclusion within the cyclodextrin cavity. Upon co-assembly with MCP and Prt to form MPCR NPs, a slight decrease in stability was observed. This reduction may be attributed to competitive hydrophobic interactions during assembly, such as those involving alanine residues in Prt or residual methoxy groups in MCP, which may lead to a slight degree of RES exposure outside the CD cavity. Nonetheless, MPCR still provided substantial protection against oxidative degradation compared to free RES. Given that hydroxyl radicals (•OH) are among the most reactive and damaging ROS under pathophysiological conditions, ESR analysis was performed to assess the •OH-scavenging capacity (Fig. 2I). Free RES showed potent activity, with a scavenging efficiency of 82%, whereas CR exhibited a reduced effect, likely due to steric hindrance from encapsulation. In comparison, MPCR NPs achieved a higher scavenging efficiency of 75%, which may be attributed to the additive antioxidant effects of both MCP and CR. These results suggested that the MPCR nanostructure confers protective effects, enhancing RES molecular stability while maintaining its biological activity. Collectively, these results demonstrated that MCP can be assembled into a charge-driven NP platform capable of integrating hydrophobic drug–carrier complexes while conferring protective effects that enhance molecular stability and preserve biological activity, highlighting its potential for modular therapeutic design.

### Structural and spectroscopic characterization

In Figs. 3A and S5, FTIR spectra revealed significant differences between CP and MCP, corresponding to chemical transformations induced by alkaline de-esterification and sodium ion exchange. In MCP, the saturated C–H stretching band at 2940 cm^− 1^, prominent in CP, was markedly reduced, likely due to the removal of alkyl groups such as methoxy moieties, resulting in fewer C–H bonds. The strong ester carbonyl stretching at 1750 cm^− 1^, assigned to methyl ester groups (-COOCH_3_), completely disappeared in MCP, confirming effective de-esterification. Concurrently, COO⁻ asymmetric stretching at 1630 cm^− 1^ observed in CP was replaced by a broad band centered at 1615 cm^− 1^ in MCP, indicative of the asymmetric stretching of carboxylate sodium salts (-COONa), reflecting an altered electron density and bonding configuration post-ionization. Further changes were observed in the 1444–1240 cm^− 1^ region. In CP, this region featured multiple overlapping peaks of comparable intensities, mainly attributed to C–O–C ether stretching and C–OH bending vibrations associated with side-chain saccharide structures. In contrast, MCP exhibited three distinct peaks at 1420, 1334, and 1240 cm^− 1^. The 1420 cm^− 1^ band corresponded to symmetric -COO^−^ stretching (typical for -COONa), while the 1334 and 1240 cm^− 1^ peaks were respectively assigned to C–OH bending and C–O stretching, indicating a reorganization of functional groups in both the backbone and side-chain regions. These spectral changes collectively confirmed successful de-esterification and conversion to the sodium salt form. GPC and ^1^H NMR analyses confirmed a substantial decrease in molecular weight, from native CP (485 kDa) to MCP (12.6 kDa), and in degree of esterification (from 57.86% to 7.36%) (Figs. S1, S2) [52], validating the effective preparation of MCP.

In Fig. 3A and B, the FTIR spectrum of Prt exhibited characteristic amide I and amide II bands at 1660 and 1540 cm^− 1^, respectively corresponding to C = O stretching of the peptide backbone and the coupling of N–H bending with C–N stretching. An additional band at 1452 cm^− 1^ was assigned to aliphatic C–H bending, potentially arising from methylene scissoring vibrations in arginine-rich side chains. Upon co-assembly with MCP to form MP NPs, the amide I band of Prt red-shifted from 1660 to 1656 cm^− 1^, indicating intermolecular interactions affecting peptide C = O stretching, possibly accompanied by localized secondary structure rearrangement. Simultaneously, MCP absorption bands at 1615 and 1334 cm^− 1^, respectively corresponding to asymmetric -COO^− 1^ stretching and C–OH/C–O bending, shifted to 1609 and 1329 cm^− 1^. These shifts were attributed to electrostatic and hydrogen bonding interactions between anionic MCP and cationic amine groups of Prt. Additionally, the symmetric -COO^−^ stretching of MCP shifted from 1420 to 1416 cm^− 1^, potentially reflecting Na^+^ displacement or hydrogen bond network reorganization upon complexation. Together, these systematic spectral shifts support the presence of defined electrostatic and hydrogen bonding interactions between Prt and MCP, accompanied by localized structural rearrangements that facilitate the stable formation of MP polyelectrolyte complexes.

Upon incorporation of CMCD into MP NPs to form MPC NPs, the FTIR spectrum exhibited a broadened absorption band at 1622 cm^− 1^ with a more-symmetric profile and a slight shift compared to corresponding peaks in either CMCD or MP NPs alone. These changes suggested that CMCD-modulated the original electrostatic interactions and hydrogen bonding network between -COO^−^ and amide groups in MP NPs, promoting a reorganization of the charge distribution and molecular alignment. Further incorporation of the CR complex into the assembly resulted in MPCR NPs, whose FTIR spectrum showed additional shifts in both asymmetric and symmetric -COO^−^ stretching bands to 1614 and 1422 cm^− 1^, respectively. These spectral changes indicated further refinement of the electrostatic balance and hydrogen bonding landscape upon CR integration. This trend paralleled the stepwise reduction in NP sizes observed from MP to MPC and to MPCR NPs (Fig. 2C), supporting the regulatory role of CMCD and its complex with RES (CR), in fine-tuning the nanostructure. Moreover, the aromatic C = C stretching signal of RES at 1516 cm^− 1^ remained unchanged in MPCR NPs, suggesting that RES remained stably encapsulated within the CMCD hydrophobic cavity throughout NP assembly and was not disrupted by subsequent polyelectrolyte complexation.

Circular dichroism (CD) spectroscopy further revealed the secondary structural evolution of Prt during different stages of NP assembly (Fig. 3C, Table S2). Native Prt exhibited a predominantly random coil conformation (49.6%) with a substantial proportion of a β-sheet structure (32.2%), primarily of the antiparallel subtype. Upon complexation with MCP to form MP NPs, the β-sheet content increased to 39.7%, while the random coil fraction decreased to 44.7%, suggesting that polyelectrolyte complexation with MCP stabilized previously disordered regions into more-ordered β-sheet structures. In contrast, Prt mixed with CMCD alone did not induce α-helix formation (only 0.9%) and disrupted the antiparallel-left twisted β-sheet subtype, reducing overall β-sheet content to 28.2%. This indicated that CMCD perturbed the native β-sheet architecture of Prt, in contrast to the stabilizing effect of MCP. When CMCD and MCP were co-assembled into MPC NPs, the total β-sheet content remained comparable to that of MP NPs (39.0%), but the β-sheet composition shifted toward relaxed and right-twisted subtypes. This conformational transition was accompanied by an increase in β-turns (20.7%) and a further reduction in the random coil content (37.1%), suggesting a cooperative effect between CMCD and MCP in modulating the β-sheet subtype distribution of Prt, thereby promoting a more structurally adaptive and flexible conformation. Upon incorporation of CR to form MPCR NPs, the β-sheet content markedly declined to 25.4%, while the random coil fraction increased to 61.7%, with a complete loss of α-helices and a decrease in the β-turn content. These changes indicated that the inclusion of RES altered the spatial conformation and surface characteristics of CMCD, thereby modifying its interaction with Prt. The larger and more-rigid CR complex likely introduced steric hindrance that restructured the binding interface, redistributed the contact geometry, and perturbs the previously ordered Prt conformations induced by MCP and CMCD. This shift toward a more disordered and flexible conformation promoted conformational plasticity that supported spontaneous, electrostatically driven self-assembly into stable NPs, consistent with the observed size reduction. Moreover, the increased conformational looseness may contribute to improved drug release kinetics or responsive behavior of the nanostructure.

### Colloidal stability, sequential release, and pathology-responsive behavior

**Fig. 4:**
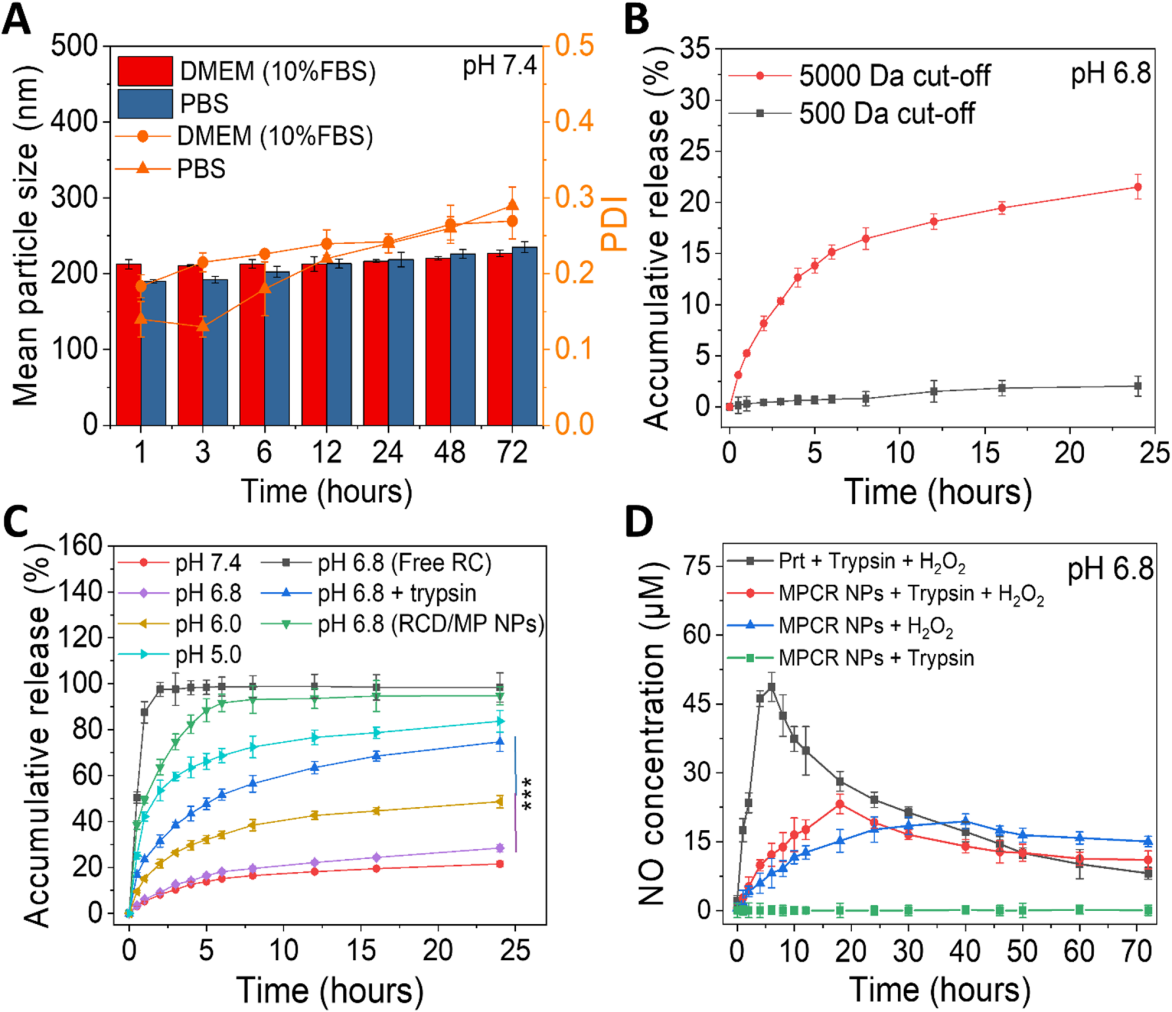
Colloidal stability, drug release, and NO generation of MPCR NPs. (**A**) Dynamic changes in size and PDI of MPCR NPs in PBS (pH 7.4, 37 °C) over 72 h (*n* = 6). (**B**) RES and CR release profiles defined by membranes with different molecular weight cutoffs in PBS (pH 6.8, 37 °C) (*n* = 6). (**C**) CR release profiles under various pH conditions with or without trypsin (2500 USP units/mg protein) in PBS (pH 7.4, 37 °C) (*n* = 6). (**D**) NO production from MPCR NPs in the presence of trypsin (2500 USP units/mg protein) and H_2_O_2_ (100 µM) in PBS (pH 6.8, 37 °C), with a Prt-equivalent concentration of 0.3 mg/mL (*n* = 6). ***p < 0.001

Following establishment of the structural and conformational characteristics, we further evaluated their colloidal stability, controlled release behavior, and functional bioactivity. In simulated physiological media, including phosphate-buffered saline (PBS; pH 7.4) and Dulbecco’s modified Eagle medium (DMEM) supplemented with 10% fetal bovine serum (FBS), MPCR NPs demonstrated high colloidal stability, with no significant changes in particle size or PDI observed over 24 h. Even after 72 h, the hydrodynamic diameter remained below 220 nm with a PDI of < 0.25 (Fig. 4A). In addition, extended storage stability evaluation revealed that MPCR NPs maintained colloidal integrity and antioxidant activity for up to 30 days under refrigerated conditions (4 °C), whereas samples stored at 25 °C exhibited progressive increases in particle size and PDI, accompanied by a pronounced reduction in hydroxyl radical scavenging capacity (Fig. S6).

To investigate the release mechanism, MPCR NPs were placed in dialysis bags with different molecular weight cutoffs to distinguish whether the released cargo was free RES or the intact CR. In the 5000-Da cutoff group, RES-associated fluorescence was detectable in the release medium, showing a sustained release profile with only 16% cumulative release after 8 h. In contrast, the 500-Da cutoff group exhibited a negligible fluorescence signal, indicating that the released species were predominantly intact CR rather than free RES (Fig. 4B). Cyclodextrin-based inclusion complexes are known to release hydrophobic guest molecules through lipid-mediated displacement or membrane interactions, where localized hydrophobic environments facilitate guest dissociation [53, 54]. Thus, the observed sequential release, initial liberation of CR into the extracellular medium followed by RES release upon membrane interaction, represents a cascade mechanism that helps minimize premature RES exposure to aqueous environments that promote oxidation or metabolic degradation, thereby preserving its molecular stability and bioactivity until reaching the cellular interface.

To mimic the pathological microenvironment of aortic lesions, characterized by mild acidity [55], elevated oxidative stress [56], and increased protease activities of trypsin and other serine proteases [57], we further evaluated the pH- and enzyme-responsiveness of MPCR NPs. As shown in Fig. 4C, MPCR NPs exhibited a clear pH-dependent release behavior, with progressively accelerated CR release under decreasing pH conditions. At pH 5.0, the release rate increased by approximately 4.2-fold compared to that at pH 7.4 over 8 h. This pH sensitivity can be attributed to protonation of the carboxyl groups on both CMCD and MCP under acidic conditions, which reduces their negative surface charge and weakens electrostatic interactions with positively charged proteins. The loss of charge-mediated retention likely contributes to matrix relaxation and facilitates CR release. Supporting this hypothesis, control NPs loaded with a neutral cyclodextrin-RES complex lacking carboxyl functionalities (CDR/MPs) exhibited a burst release profile under similar conditions. In a mildly acidic and protease-rich environment (pH 6.8 with trypsin), mimicking inflammatory vascular lesions, the presence of protease further accelerated CR release, resulting in a 2.9-fold increase by 8 h. These results demonstrated that MPCR NPs are responsive to pathological stimuli relevant to AD, underscoring their potential for site-specific drug release in inflamed vascular tissues.

Given the elevated oxidative stress in aortic lesions, we further assessed the NO, releasing capacity of MPCR NPs under simulated pathological conditions using an H_2_O_2_-containing environment. As Prt is a known NO donor [33], this experiment aimed to evaluate its release kinetics within the assembled nanostructure. As shown in Fig. 4D, free Prt exhibited a burst-like NO release profile, reaching a peak concentration of 48.7 µM at 6 h in the presence of both H_2_O_2_ and protease. In contrast, MPCR NPs produced 12.2 µM of NO at 6 h and achieved a delayed peak of 23.2 µM at 18 h, indicating a more-sustained release profile. Notably, after 50 h, the cumulative NO output from MPCR NPs surpassed that of free Prt, suggesting that the responsive nanoplatform preserved the NO-generating capacity of Prt while attenuating its burst release behavior. Furthermore, in the absence of protease, MPCR NPs maintained their structural integrity, leading to an even more prolonged and gradual NO release profile. This observation highlights the role of the nanoassembly in providing kinetic control over NO release in response to environmental stimuli. Collectively, these results demonstrated that the co-assembly of Prt, MCP, and CMCD resulted in a responsive multicomponent nanoplatform capable of pathology-triggered NO generation with temporally regulated kinetics, supporting its therapeutic potential for inflammation-associated vascular disorders such as AD.

### Cytocompatibility and spatiotemporal targeting

**Fig. 5:**
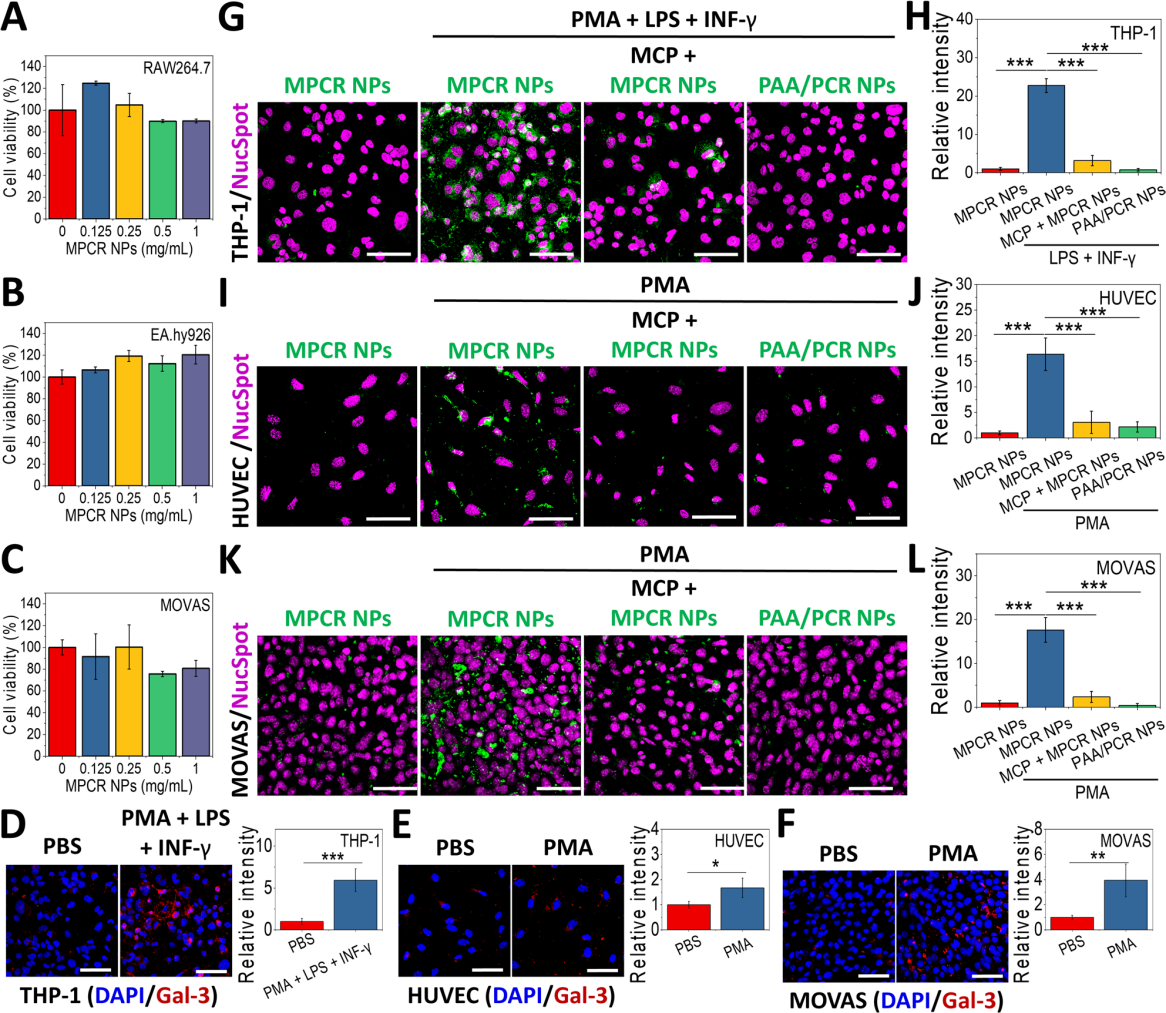
In vitro evaluation of the cytocompatibility, Gal-3 expression, and Gal-3-mediated cellular uptake of MPCR NPs. (**A**-**C**) Viability of RAW264.7, EA.hy926, and MOVAS cells after 24 h of incubation with MPCR NPs (0–1 mg/mL). (**D**-**F**) Immunofluorescence analysis of Gal-3 expression (red) in THP-1-derived M1 Mφs, inflamed HUVECs, and inflamed MOVAS cells after proinflammatory stimulation. (**G**-**L**) Representative images showing cellular uptake (green) of FL-labeled MPCR NPs in different cell types under inflamed conditions, with or without MCP blocking or replacement with PAA/PCR NPs. Nuclei were stained with NucSpot^®^ (magenta). Quantification of fold changes of cellular uptake relative to the controls (*n* = 4). Scale bars: 50 μm. * *p* < 0.05, ** *p* < 0.01, *** *p* < 0.001

The cytocompatibility of MPCR NPs was evaluated using key cell types involved in AD progression, including Mφs (RAW264.7), ECs (EA.hy926), and VSMCs (MOVAS), to ensure biosafety. As shown in Fig. 5A and C, MPCR NPs exhibited good biocompatibility at concentrations of up to 1 mg/mL, which can be attributed to their main components, all of which are known for their high biosafety. Following confirmation of the cytocompatibility, the cellular association of MPCR NPs was further examined to assess their affinity toward key pathological cell types involved in AD. Upon exposure to appropriate pro-inflammatory stimuli, Mφs (THP-1-derived M1 phenotype), inflamed ECs (phorbol 12-myristate 13-acetate (PMA)-stimulated HUVECs), and inflamed VSMCs (PMA-stimulated MOVAS cells) exhibited markedly upregulated Gal-3 expression (Fig. 5D-F), with respective 5.4-, 1.8-, and 3.8-fold increases, relative to their unstimulated controls. Correspondingly, fluorescence-labeled MPCR NPs exhibited a 22.8-fold increase in uptake by stimulated THP-1 cells (Fig. 5G, H). Notably, pre-blocking Gal-3 with free MCP, removing MCP from MPCR NPs (replacing it by PAA; PAA/PCR NPs) (Table S1), or silencing Gal-3 expression via siRNA (Fig. S7) significantly reduced NP uptake, highlighting the Gal-3-mediated specificity rather than nonspecific endocytosis. Similar trends were observed in stimulated HUVECs and MOVAS cells, with 16.4- and 17.6-fold respective increases in uptake (Fig. 5I-L). These results emphasize the pathology-tailored delivery capability of Gal-3-targeted systems within inflamed vascular microenvironments.

**Fig. 6:**
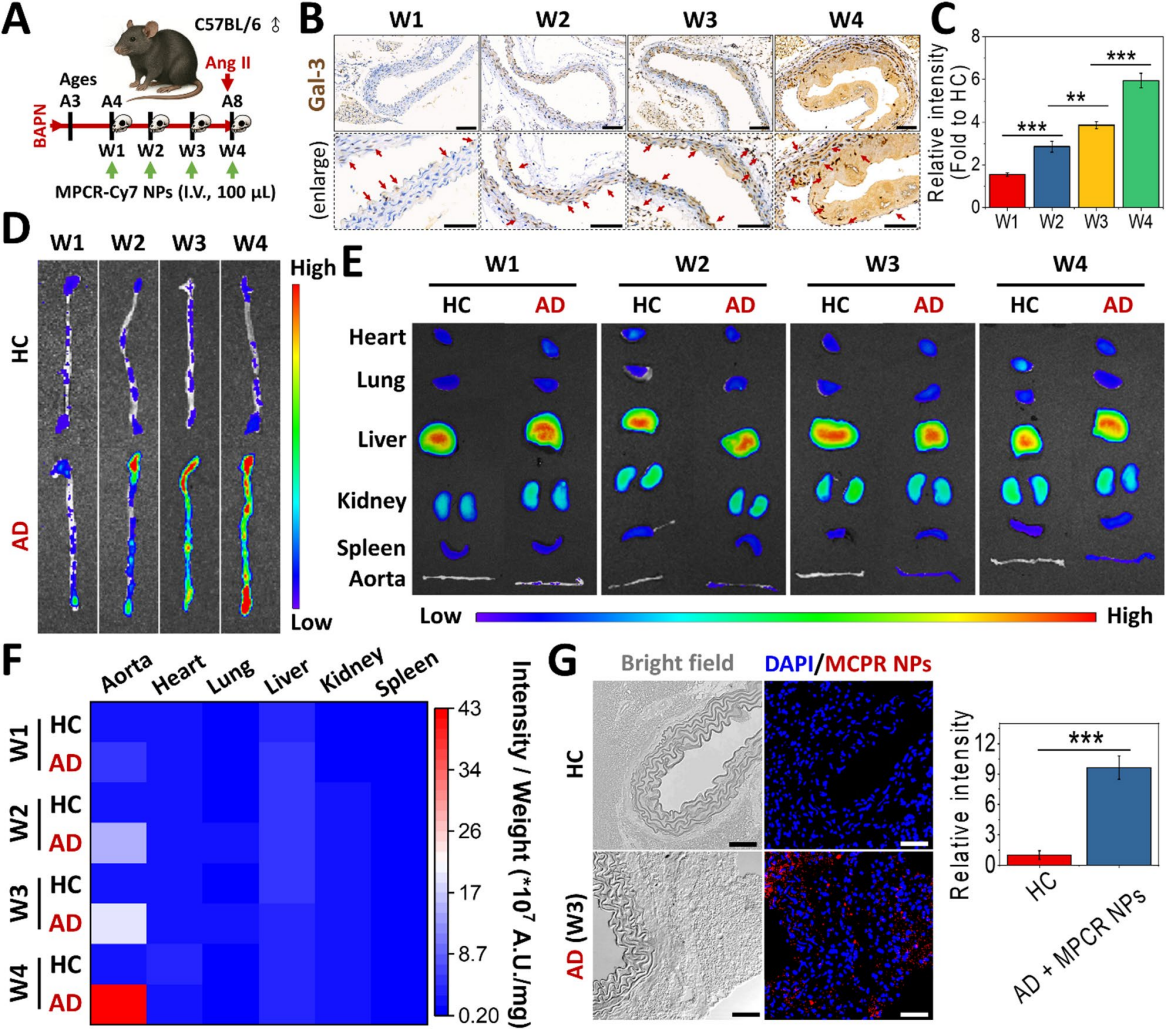
Targeting and biodistribution of MPCR NPs in an in vivo AD model. (**A**) Schematic of the BAPN/Ang II-induced AD mouse model and treatment timeline. (**B**) IHC staining of Gal-3 in aortic tissues from AD mice (scale bars: 100 μm; red arrows indicate representative Gal-3-positive regions). Images outlined with dashed boxes represent magnified views of selected regions. (**C**) Quantitative analysis of Gal-3 expression (*n* = 3). (**D**) IVIS imaging of aortas and (**E**) biodistribution analysis at 6 h post-injection of Cy7-labeled MPCR NPs in AD mice at different stages (W1–W4); HC, healthy control. (**F**) Quantitative fluorescence intensity analysis using IVIS imaging software. (**G**) CLSM images of cryosectioned aortic tissues (W3) showing Cy7-labeled MPCR NP deposition (red) and nuclei (DAPI, blue) within AD lesions. Scale bars: 25 μm. Quantitative analysis (*n* = 3). ** *p* < 0.01, *** *p* < 0.001

A more-comprehensive evaluation of the targeting capacity was conducted using in vivo AD models representing different disease stages. Continuous ad libitum administration of BAPN in drinking water was used to compromise the structural integrity of the aortic wall, inducing mild aortic dilation, fiber disruption, and elastin fragmentation, thereby establishing an early-stage or subclinical form of AD. At week 4, subcutaneous implantation of an ALZET^®^ osmotic pump delivering angiotensin (Ang) II was performed to exacerbate hemodynamic stress and the inflammatory burden, ultimately progressing to a dissection/rupture model (Fig. 6A). During the 4-week induction phase (W1-W4), Gal-3 expression was first detected in the tunica intima at W1 following 1 week of BAPN exposure. By W2, Gal-3 levels had significantly increased and had partially extended into the tunica media. At W3, expression had further increased and become broadly distributed throughout the vessel wall, and Gal-3-positive cells were also observed in the perivascular connective tissue, suggesting an early adventitial inflammatory response. By W4, overt intimal tearing and the formation of a false lumen were observed, with Gal-3 remaining highly expressed across the vessel wall and within the false lumen (Fig. 6B, C). These results demonstrated the spatiotemporal persistence of Gal-3 expression from early to late stages of AD, and its strong association with key pathological cell types, ranging from intimal ECs and medial VSMCs to Mφs residing in the adventitia and false lumen, throughout disease progression.

To evaluate the in vivo targeting performance of MPCR NPs, fluorescence imaging was performed using an in vivo imaging system (IVIS) in AD mice at different disease stages, following cyanine 7 (Cy7) labeling of the NPs. Compared to healthy controls (HCs), significantly higher aortic accumulation of MPCR NPs was observed as early as W1 (2.66-fold), and this difference sharply increased over time, reaching 6.41-fold at W2, 9.85-fold at W3, and 19.95-fold at W4 (Fig. 6D). This trend of stage-dependent accumulation closely matched the Gal-3 expression profile across AD progression (Fig. 6B, C), supporting the Gal-3-targeted mechanism as a key driver of pathology-tailored precision delivery. Compared to existing AD-targeted nanoplatforms, this Gal-3-guided strategy demonstrated superior sensitivity and early-stage responsiveness. For example, a neutrophil-mimetic delivery system that relies on neutrophil chemotaxis toward inflammatory vascular lesions showed an approximately 1.5-fold increase in aortic accumulation only at the mid-to-late stage of AD (3 weeks after Ang II + BAPN co-induction) [7]. Another approach targeting the αvβ3 integrin, upregulated in inflamed and angiogenic endothelium and medial VSMCs, showed detectable aortic accumulation starting at W2 after BAPN induction [6]. While differences in fluorescent dyes and incomplete reporting of control groups in previous IVIS studies limit direct numerical comparisons, the available data still highlight the advantage of the Gal-3-targeting strategy, as embodied by MPCR NPs, in achieving earlier and more-robust targeting in AD.

In addition to aortic accumulation, the biodistribution profile of MPCR NPs was assessed across major organs to evaluate off-target localization. As shown in Fig. 6E, the liver exhibited relatively high fluorescence, which may have resulted from both its role in NP clearance via the reticuloendothelial system and its large tissue mass, while the kidneys, spleen, and lungs showed minimal signals [58]. To determine whether these visual differences reflected the actual NP distribution, fluorescence intensities were further normalized to tissue weights (Fig. 6F). The resulting heatmap revealed a marked and progressive increase in aortic accumulation from W1 to W4, with the aorta emerging as the dominant site of MPCR NP deposition by W4. Notably, the aortic signal at W4 was approximately 10-fold higher than that of the liver, underscoring the strong pathological selectivity of the system. Aortic cryosections further confirmed tissue-level localization of MPCR NPs. In W3 AD mice, a strong Cy7 signal was detected within the vessel wall and surrounding perivascular regions (Fig. 6G), with a 9.6-fold higher intensity than that observed in healthy controls, where the signal was negligible. These results provide direct histological evidence of inflammation-associated accumulation, consistent with regions of Gal-3 upregulation at this stage.

To investigate Gal-3 expression at the cellular level in AD lesions, we performed quadruple immunofluorescence staining on aortic sections from W3 AD mice. Confocal images revealed prominent Gal-3 expression in CD31^+^ ECs, α-SMA^+^ VSMCs, and CD68^+^ Mφ. These findings provide direct spatial and cell-type-level evidence that Gal-3 is concurrently upregulated in multiple pathological cell types during the pre-rupture stage of AD. This result offers compelling validation for the central design logic of our nanoplatform, which leverages Gal-3 as a unified molecular target to achieve multi-cellular targeting within the inflamed aortic wall. Unlike conventional vascular markers that emerge only after significant tissue damage, Gal-3 exhibits early, sustained, and multi-compartment expression, making it an attractive and actionable target for pathology-tailored intervention.

**Fig. 7:**
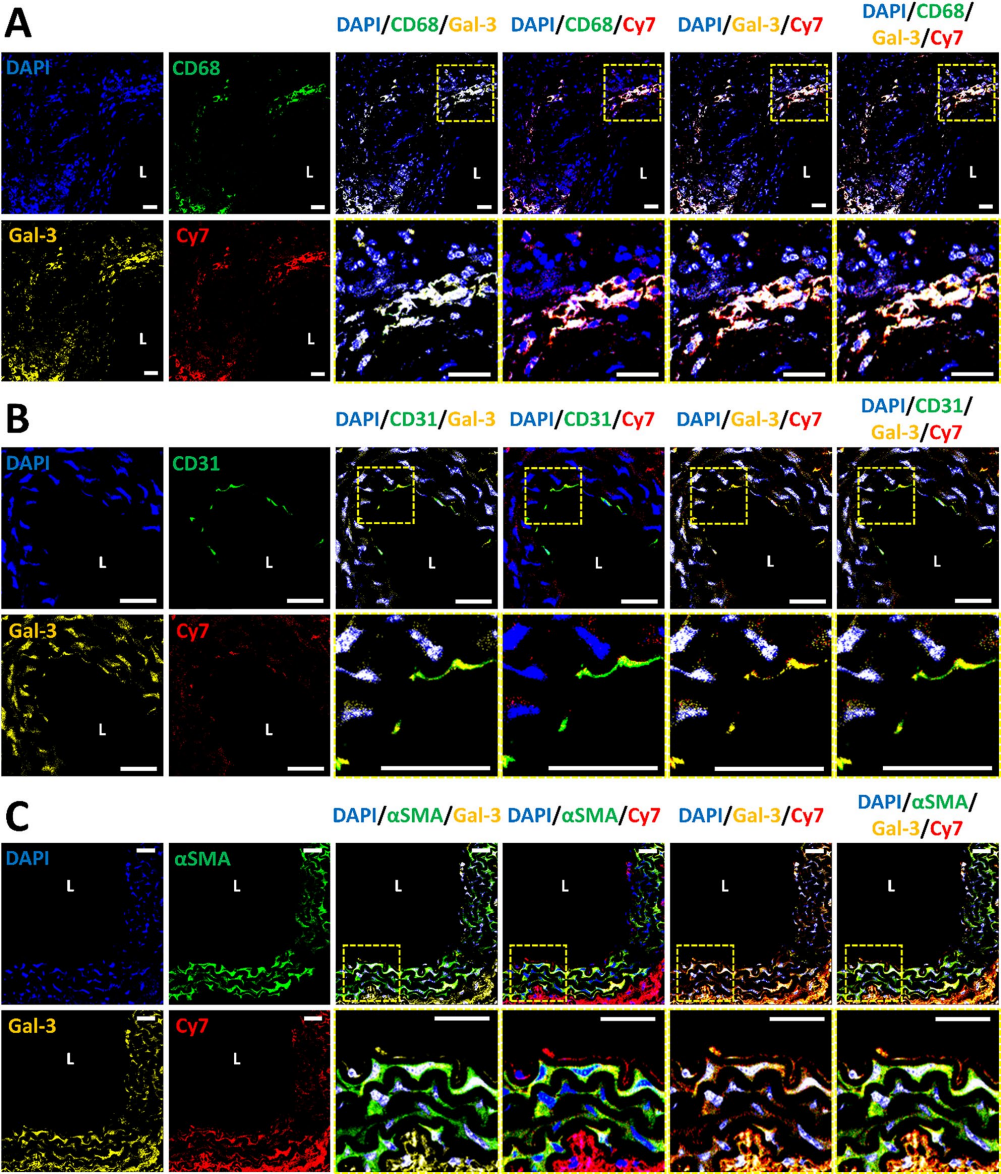
Confocal laser scanning microscopy (CLSM) images of aortic sections from AD mice at week 3. Sections were stained with DAPI (nuclei, blue), Gal-3 (yellow), cell-type markers (green: CD68 for Mφ (**A**), CD31 for ECs (**B**), or α-SMA for VSMCs (**C**)), and Cy7-labeled MPCR NPs (red). Dashed boxes indicate the regions selected for high-magnification display. Scale bars: 25 μm. L: lumen

To further evaluate the in vivo localization of MPCR NPs, we examined the spatial relationship between Cy7-labeled NPs and Gal-3 expression. Cy7 fluorescence colocalized with Gal-3 signals across all layers of the vessel wall, indicating Gal-3-mediated enrichment of MPCR NPs in inflamed regions. Furthermore, MPCR NPs were found to overlap with each of the three Gal-3^+^ cell types, including ECs, VSMCs, and Mφ. These results provide direct in vivo evidence of multi-cellular targeting enabled by Gal-3 recognition, reinforcing the mechanistic foundation and translational potential of our pathology-driven delivery strategy (Fig. 7).

### Pathology-tailored multi-functional therapeutic approach

**Fig. 8:**
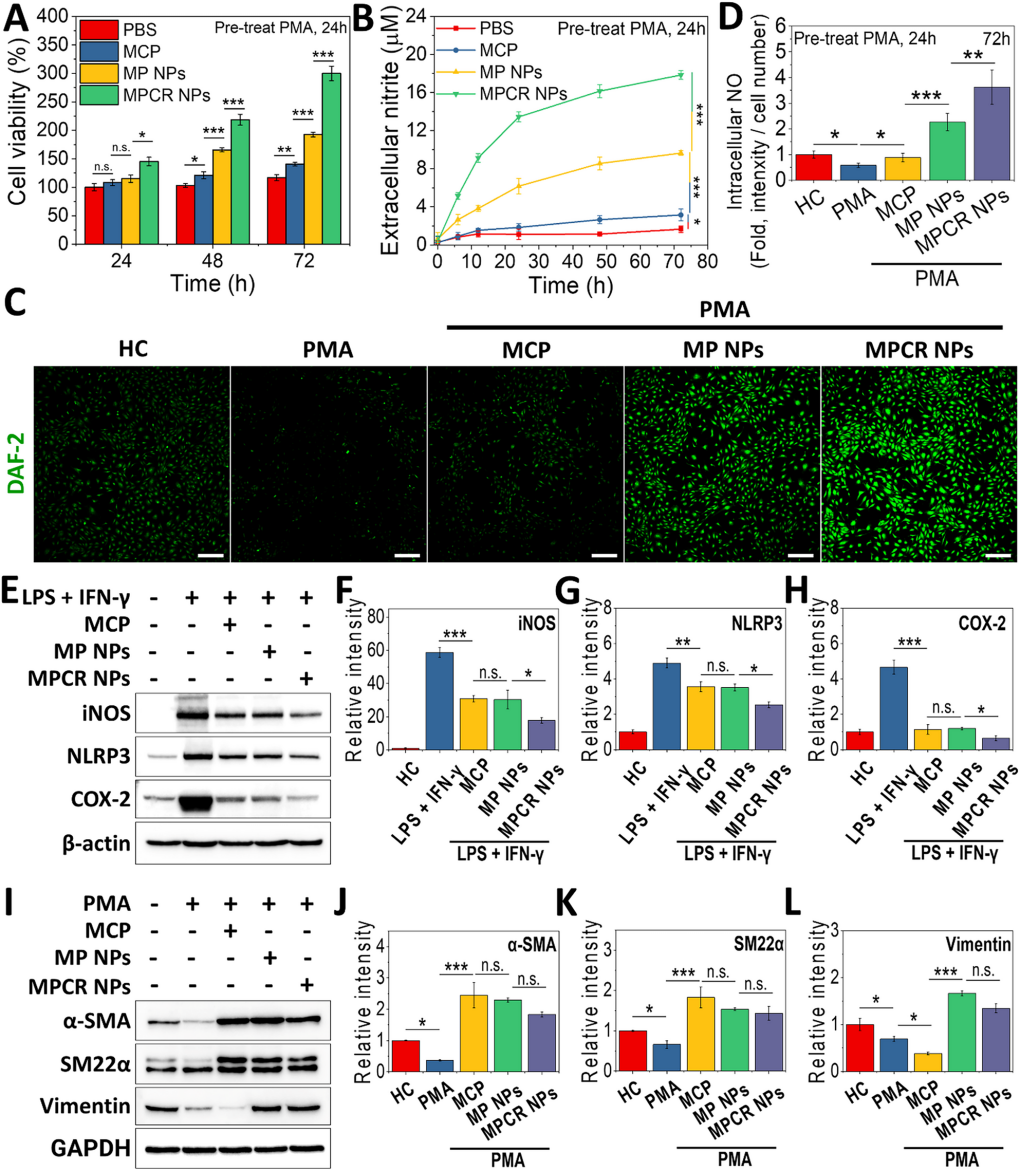
NO production and ECs proliferation. Cell viability (**A**), NO release (**B**), and intracellular NO accumulation (**C**, **D**) of treatments (0.5 mg/mL MPCR NPs; Equiv. 44.5 µg Prt/mL) after 24 h of co-incubation with 100 nM PMA (*n* = 6). Scale bar: 200 μm. (**E**-**H**) Western blot and quantitative analysis of iNOS, NLRP3, and COX-2 in Mφs (*n* = 3). (**I**-**L**) Western blot and quantitative analyses of α-SMA, SM22α, and vimentin in VSMCs (*n* = 3). The corresponding uncropped Western blot images with molecular weight markers are shown in the Supporting Information as Figs. S10, S11. n.s., non-significant difference, * *p* < 0.05, ** *p* < 0.01, *** *p* < 0.001

Building upon the demonstrated targeting capability of MPCR NPs, we next evaluated their functional therapeutic potential in key pathological cell types implicated in AD progression, including ECs, Mφs, and VSMCs. After PMA treatment, ECs viability remained suppressed over 3 days (Fig. 8A), indicating near-complete arrest of proliferation. During this period, NO production was markedly reduced (Fig. 8B). MCP treatment resulted in a modest improvement, increasing NO production by 1.9-fold, likely due to its anti-inflammatory effects and inhibition of Gal-3, which suppresses eNOS expression and activity through inflammatory signaling pathways and oxidative stress [59]. Blocking Gal-3 may therefore help restore eNOS function and enhance endogenous NO production. In contrast, combining MCP with Prt to form MP NPs led to a significant 3.1-fold increase in NO production compared to MCP alone. Concurrently, intracellular NO accumulation (Fig. 8C, D) and cell viability (Fig. 8A) substantially improved, suggesting that MP NPs, as a sustained nano-scale NO donor (Fig. 4D), effectively ameliorated endothelial dysfunction. Notably, MPCR NPs, which co-deliver RES, further increased NO levels by 1.9-fold compared to MP NPs and enhanced cell viability. These results highlight the potent therapeutic synergy between a sustained NO supply and the multifaceted protective actions of RES, including reduction of apoptosis, upregulation of eNOS expression, and correction of eNOS uncoupling [60], together achieving comprehensive restoration of endothelial function. In addition, the observed increase in both intracellular and extracellular NO levels upon RES-containing treatment (Fig. 8B–D) may also reflect SIRT1-mediated eNOS activation, a well-established pathway by which RES promotes endothelial NO bioavailability [39, 40]. Given the documented role of SIRT1 in modulating mitochondrial oxidative stress through activation of PGC-1α and FOXO3a, the enhanced NO signaling may further contribute to a reduction in mtROS and preservation of endothelial homeostasis.

Mφ-mediated inflammation is a critical driver of AD progression, involving upregulation of key inflammatory mediators such as inducible NO synthase (iNOS), NLRP3 inflammasome components, and cyclooxygenase-2 (COX-2). These molecules contribute to oxidative stress, cytokine release, and ECM degradation through pathways including NF-κB activation and NLRP3 inflammasome assembly, ultimately exacerbating vascular injury and remodeling [61]. In Figs. 7F and 8E, under lipopolysaccharide (LPS) and IFN-γ stimulation, Mφs exhibited significantly elevated expression of iNOS, COX-2, and NLRP3, confirming successful induction of a pro-inflammatory phenotype. MCP treatment led to significant reductions in expressions of iNOS (1.9-fold), NLRP3 (1.4-fold), and COX-2 (4.1-fold), compared to the LPS/IFN-γ group, indicating potent anti-inflammatory activity (Fig. 8F-H). This effect was likely mediated by MCP’s inhibition of Gal-3, which promotes NLRP3 inflammasome assembly and upregulates iNOS and COX-2 expression via activation of NF-κB and related inflammatory signaling pathways [62]. MP NPs exhibited minimal additional reduction relative to MCP, suggesting that sustained NO release alone did not further suppress inflammasome activation or inflammatory mediator expression beyond the effect of Gal-3 blockade. In contrast, MPCR NPs further reduced the expression of iNOS (1.8-fold), NLRP3 (1.4-fold), and COX-2 (1.8-fold), compared to the MP NPs group (Fig. 8F-H). This enhanced suppression reflects the mechanistic synergy between MCP and RES, where MCP inhibits Gal-3-mediated priming of the inflammasome and RES targets downstream events by preserving mitochondrial integrity and stimulating autophagy, particularly mitophagy, which facilitates the removal of damaged mitochondria, limits mitochondrial ROS accumulation, and thereby suppresses NLRP3 activation. Together, this dual targeting of upstream priming and downstream activation nodes achieves more-effective attenuation of Mφ-driven inflammation, highlighting the potential of MPCR NPs as a pathology-tailored anti-inflammatory strategy in AD therapy [63].

**Fig. 9:**
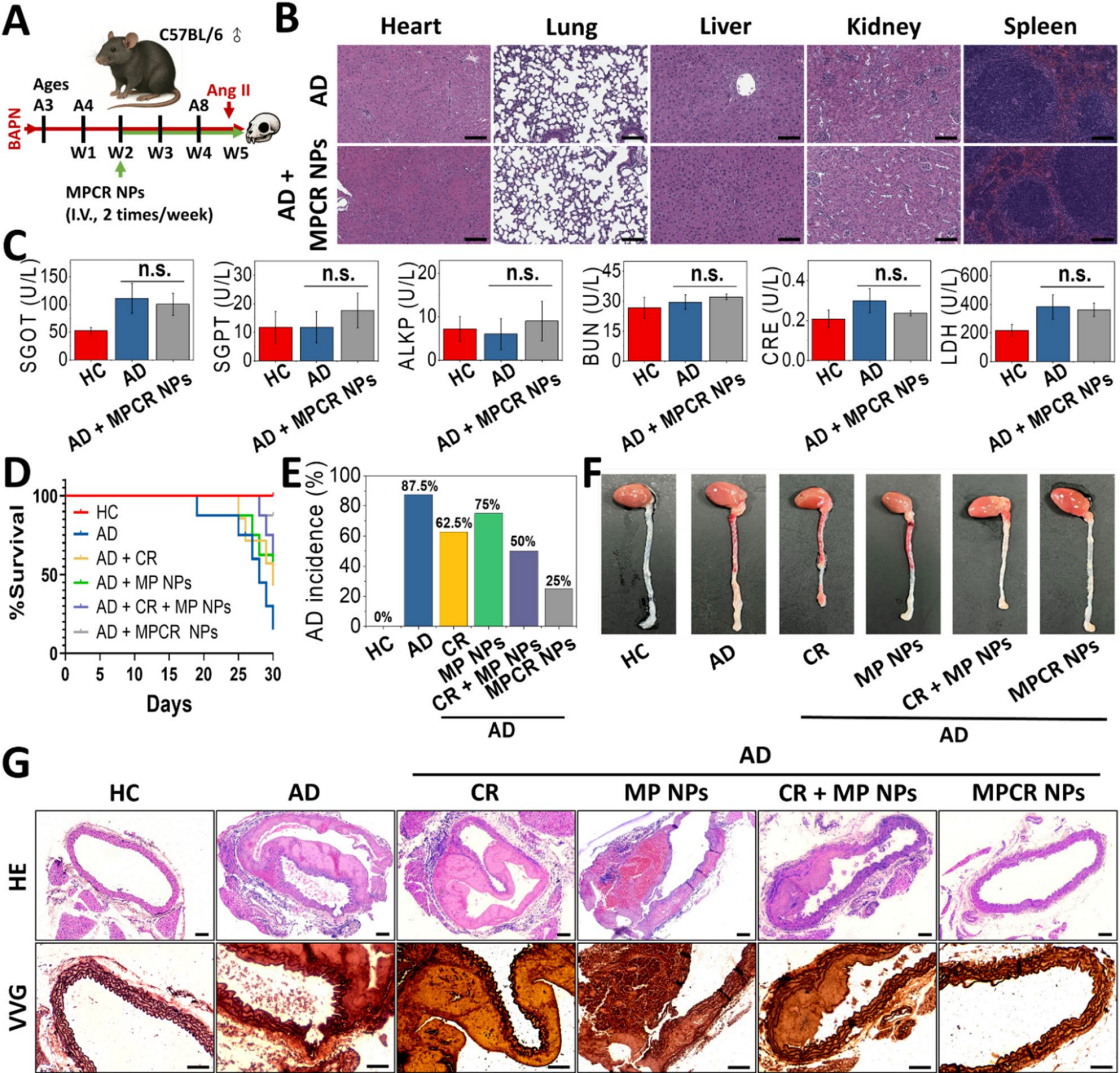
Biosafety and therapeutic efficacy of MPCR NPs in the AD mouse model. (**A**) Schematic of the treatment protocol in the BAPN/Ang II-induced AD model. All groups received intravenous injections (200 µL per dose, twice weekly for 3 weeks), corresponding to an approximate RES dose of 2.58 mg/kg per injection. (**B**) Representative H&E staining of major organs after 4 weeks of treatment (scale bars: 100 μm). (**C**) Serum biochemical parameters, including SGOT, SGPT, ALKP, BUN, CRE, and LDH, after 4 weeks of treatment (*n* = 6). (**D**) Kaplan-Meier survival curves (*n* = 8). Log-rank test p-values for comparisons with the AD group: AD vs. AD + RES (*p* = 0.2806), AD vs. AD + MCP (*p* = 0.1759), AD vs. AD + RES + MCP (*p* = 0.0333), AD vs. AD + MPCR NPs (*p* = 0.0050). (**E**) Incidence of aortic rupture (*n* = 8). Fisher’s exact test p-values for comparisons with the AD group: AD vs. AD + RES (*p* = 0.5692), AD vs. AD + MCP (*p* = 1.000), AD vs. AD + RES + MCP (*p* = 0.2821), AD vs. AD + MPCR NPs (*p* = 0.0406). (**F**) Representative images of aortas post-treatment. (**G**) H&E staining and (**H**) Verhoeff-Van Gieson (VVG) staining of aortic tissues. Scale bars: 100 μm. n.s., non-significant difference

In AD pathology, maladaptive phenotypic modulation of VSMCs plays a crucial role in pathological vessel wall remodeling and medial weakening. Among key markers, α-SMA and SM22α are hallmarks of the contractile phenotype, indicating differentiated and functionally stable VSMCs [64]. In contrast, vimentin and osteopontin (OPN) are associated with phenotypically modulated VSMCs exhibiting enhanced plasticity, migratory behavior, or synthetic activity, reflecting a shift away from the contractile state toward a secretory phenotype [65]. In Fig. 9I, upon PMA stimulation, α-SMA, SM22α, and vimentin were all markedly downregulated, reflecting global phenotypic disruption and cytoskeletal disassembly of VSMCs under inflammatory stress, rather than a directed transition toward a synthetic phenotype. Consistently, OPN expression was strongly upregulated (Fig. S8), indicating activation of the synthetic phenotype.

In the MCP treatment group, α-SMA and SM22α levels were significantly elevated, by 6.6- and 2.8-fold, respectively, compared to the PMA group (Fig. 9J, K), suggesting that preservation of the contractile phenotype may be attributed to the inhibition of Gal-3–induced phenotypic switching [66]. Vimentin expression also showed a significant 1.8-fold decrease, whereas OPN levels remained comparable to the PMA group (Fig. 9L, S8). These results suggest that MCP primarily restores contractile identity but has minimal effect on suppressing synthetic phenotype markers such as OPN. Notably, the biological significance of vimentin regulation appears to be context-dependent, varying with the phenotypic state of VSMCs [67]. Under inflammatory stress, the concurrent downregulation of α-SMA and vimentin, as observed in the PMA group, likely reflects cytoskeletal disorganization and impaired contractile and structural integrity of VSMCs [68, 69]. In contrast, MCP-treated cells, which exhibited elevated α-SMA and SM22α levels alongside reduced vimentin expression, may represent a stabilized contractile phenotype with reduced cytoskeletal plasticity and diminished responsiveness to inflammatory cues [64, 67, 69].

In the MP group, treatment with MP NPs resulted in only modest changes in α-SMA and SM22α expression compared to the MCP group, whereas vimentin expression was markedly upregulated. Interestingly, OPN expression was significantly reduced relative to the MCP group (Fig. S8), suggesting that sustained NO release more effectively suppresses synthetic phenotype acquisition. This phenotypic pattern, characterized by elevated vimentin and suppressed OPN, differs from the contractile stabilization observed with MCP alone, and may instead represent a reparative or stress-adaptive state, wherein contractile identity is partially retained while cytoskeletal plasticity is enhanced. The additional presence of sustained NO release in the MP group may have contributed to vimentin upregulation through activation of cytoskeletal remodeling pathways. Although direct regulation of vimentin by NO remains unclear, NO is known to modulate cytoskeletal dynamics and cellular migration in VSMCs, processes closely associated with intermediate filament reorganization [69, 70]. This interpretation aligns with the known role of vimentin in cytoskeletal adaptation under mechanical or inflammatory stress [69], and with the association between α-SMA/SM22α expression and contractile identity stabilization [64]. This state may support both vascular stabilization and adaptive remodeling, suggesting that MP NPs, by synergistically providing Gal-3 inhibition and sustained NO release, facilitate not only anti-inflammatory effects but also functional phenotype reprogramming toward vascular repair.

In the MPCR group, treatment with MPCR NPs resulted in slight, non-significant decreases in α-SMA and SM22α expression compared to the MP NPs group, while vimentin levels remained largely unchanged. This outcome suggests that RES had a limited capacity to further enhance preservation of contractile markers in the context of the already strong synergistic effects of MCP and NO, which act through distinct yet complementary pathways. Notably, MPCR NPs significantly reduced OPN expression compared to MP NP groups (Fig. S8), suggesting that RES may primarily contribute to the suppression of synthetic phenotype acquisition rather than enhancement of contractile identity. This selective effect on synthetic markers complements the contractile phenotype stabilization provided by MCP and NO. Importantly, MPCR NPs still demonstrated substantial improvement compared to the PMA-stimulated group, increasing α-SMA and SM22α expression by 5.0- and 2.2-fold, respectively, and even exceeding levels in the unstimulated control group by 1.8- and 1.4-fold. Overall, these findings indicated that MCP and NO exerted a clear synergistic effect in preserving key contractile markers, while RES further enhances suppression of synthetic markers. Together, these components endow MPCR NPs with strong therapeutic potential for stabilizing the VSMC phenotype and mitigating vascular remodeling. Other inflammatory stimuli, such as Ang II, have also been shown to induce Gal-3 expression in VSMCs [20, 22], underscoring its relevance in inflammation-associated vascular remodeling.

Collectively, these results demonstrate that the pathology-tailored nanoplatform based on MPCR NPs enables effective multi-target modulation across key pathological cell types involved in AD progression. Through the synergistic actions of MCP-mediated Gal-3 inhibition, sustained NO release, and RES co-delivery, the MPCR NP platform enhanced endothelial repair, suppressed Mφ-driven inflammation, and preserved the contractile phenotype of VSMCs, supporting its potential as a multifunctional nanotherapeutic strategy for the treatment of AD.

### In vivo validation of pathology-tailored nanotherapy

While the in vitro results confirmed the Gal-3-mediated cellular affinity and multi-synergistic therapeutic potential of MPCR NPs, it remained necessary to determine whether their targeted and pathology-responsive features could translate into therapeutic benefit and biosafety in vivo. To this end, we proceeded with validation studies in a BAPN + Ang II-induced AD mouse model. After 4 weeks of continuous MPCR NP treatment, we evaluated the biosafety using serum biochemical markers, including SGOT, SGPT, ALKP, BUN, LDH, and CRE. None of these indicators showed significant alterations following MPCR NPs administration, suggesting no evidence of hepatic, renal, or systemic toxicity (Fig. 9B). Histological examination of major organs (Fig. 9C) further confirmed the absence of morphological abnormalities in the heart, lung, liver, spleen, and kidney between the AD and MPCR NP-treated groups.

To further evaluate the in vivo behavior of MPCR NPs, we assessed the pharmacokinetic profile of RES in AD mice. As shown in Fig. S9, the plasma RES concentration in the CR group declined rapidly, with levels dropping below 5 µg/mL within 3 h and becoming nearly undetectable by 24 h. In contrast, MPCR NPs markedly extended RES circulation, maintaining plasma concentrations above 9 µg/mL at 6 h and approximately 2 µg/mL at 24 h. These results demonstrate that the MPCR nanoplatform effectively improves the systemic stability and circulation time of RES, thereby providing a pharmacokinetic basis for sustained therapeutic activity.

Encouraged by the favorable safety profile and the enhanced pharmacokinetic performance, we next examined whether MPCR NPs could alter AD outcomes in vivo. All treatment formulations were administered via tail vein injection at 200 µL per dose, twice weekly, with each group receiving equivalent concentrations of RES, MCP, and Prt as in the MPCR NP formulation. As shown in Fig. 9D, the AD group exhibited a markedly reduced survival rate of 15% over the 4-week disease induction period. Treatment with CR or MP NPs alone led to moderate improvements, raising survival to 43% and 50%, respectively. Notably, MPCR NP treatment further improved survival to 88%, highlighting the protective potential of MPCR NP treatment. The AD incidence exhibited a similar trend (Fig. 9E). In the AD group, 87.5% developed dissection, whereas CR and MP NP treatment reduced these to approximately 62.5% and 75%, respectively. The physical combination of CR and MP NPs further lowered the incidence to 50%, while MPCR NPs further reduced the incidence to below 25%, indicating a pronounced protective effect. Representative gross morphologies of the thoracic and abdominal aorta are shown in Fig. 9F. The HCs exhibited smooth, translucent vessels without signs of dilation or rupture. In contrast, the AD group showed pronounced aortic wall thickening, hematoma formation, and visible tearing along the vessel. Treatment with CR, MP NPs, or their physical combination led to partial improvements, although segmental thickening and discoloration remained evident. Notably, although CR or MP NP monotherapy provided limited benefits, combining both agents (CR + MP NPs) offered greater protection, indicating a synergistic effect involving RES administration, NO supply, and Gal-3 sequestration. Furthermore, co-delivery via MPCR NPs further enhanced therapeutic outcomes, underscoring the unique therapeutic advantage of a Gal-3-targeted strategy in the MPCR NP nanoplatform (Figs. 5 and 6) and controlled release (Fig. 4). Collectively, these results demonstrated the therapeutic potential of MPCR NPs in improving survival, reducing dissection incidence, and preserving the aortic structure in AD.

To further elucidate these findings, we next assessed histological changes using hematoxylin and eosin (H&E) staining and Verhoeff-Van Gieson (VVG) staining, by focusing on the cellular architecture and elastic fiber integrity in the aortic wall across groups. In Fig. 9G, H&E staining revealed that the AD group exhibited pronounced medial degeneration, disruption of the vessel wall structure, substantial inflammatory cell infiltration, and prominent formation of hematoma and false lumen. Treatment with CR or MP NPs alone showed only modest improvements, with persistent structural disorganization and partial reduction of inflammatory infiltration. In contrast, the combination of CR and MP NPs resulted in greater preservation of the medial architecture and a reduced false lumen area. Remarkably, MPCR NP-treated aortas displayed nearly normal histological features, with uniform wall thicknesses, minimal inflammation, and preserved smooth muscle cell alignment. VVG staining showed similar trends in the elastic fiber integrity. The AD group demonstrated fragmented and disrupted elastic lamellae, while CR or MP NP treatment partially restored elastic fiber continuity. The CR + MP NP group exhibited further improvements, yet residual disorganization was still apparent. Strikingly, MPCR NP-treated aortas displayed intact, continuous elastic fibers comparable to healthy controls. These results suggested that MPCR NPs not only effectively preserved the aortic wall integrity and elastic structure in AD, but also highlighted the advancement of MP NPs as a drug delivery platform contributing to improved therapeutic outcomes.

**Fig. 10:**
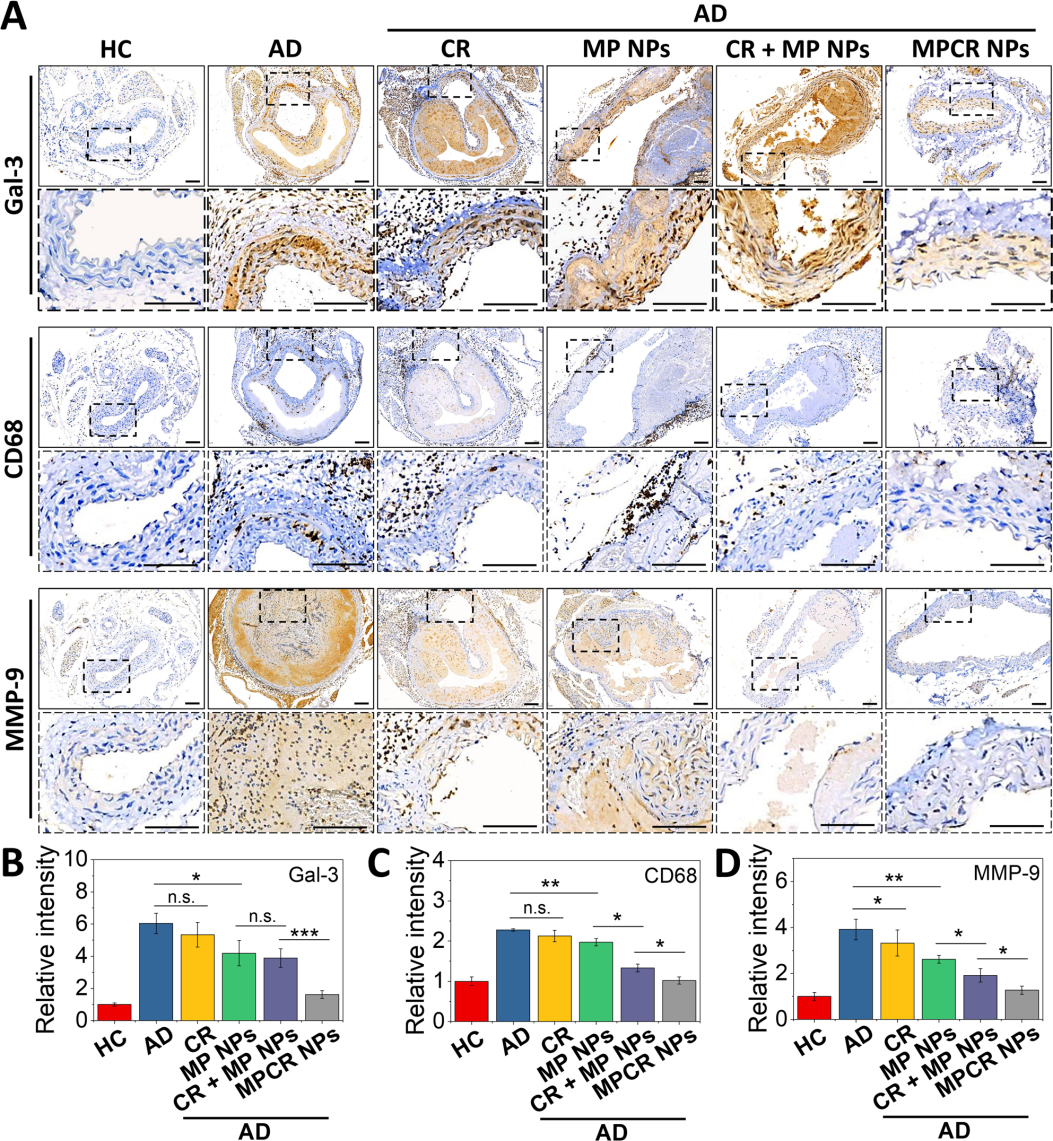
IHC staining. (**A**) IHC staining of Gal-3, CD68, and MMP-9 in aortic tissues. Images outlined with dashed boxes represent magnified views of selected regions. Scale bars: 100 μm. Dashed boxes indicate the regions selected for high-magnification display. All IHC stains were performed on serial sections from the same samples. Due to tissue heterogeneity and structural disruption in advanced AD lesions, adjacent sections may exhibit notable morphological differences. (**B**, **C**) Quantitative analysis (*n* = 3). n.s., non-significant difference, * *p* < 0.05, ** *p* < 0.01, *** *p* < 0.001

Building upon these findings, we next examined the expression of key pathological markers by immunohistochemistry, focusing on Gal-3, CD68, and MMP-9 [12]. These markers are known to drive AD pathology by promoting inflammatory cell recruitment (Gal-3), Mφ infiltration (CD68), and extracellular matrix degradation (MMP-9). In the AD group, strong IHC signals were observed throughout the endothelial, medial, and adventitial layers of the aortic wall (Fig. 10A). In particular, CD68^+^ Mφ were widely distributed across all layers of the vessel wall, reflecting pervasive inflammatory infiltration. Following treatment, especially in the CR + MP NPs and MPCR NPs groups, CD68 staining progressively retreated from the medial and adventitial layers, indicating effective suppression of Mφ-mediated inflammation. Consistent with these spatial changes, quantitative analysis revealed that MPCR NPs markedly reduced the expression of all three markers to near-baseline levels. While CR and MP NPs showed only partial reductions, the CR + MP NPs group exhibited a synergistic decrease, though not to the extent observed with MPCR NPs. These results highlight the ability of MPCR NPs to suppress upstream pathological mediators involved in dissection progression (Fig. 10B-D).

**Fig. 11:**
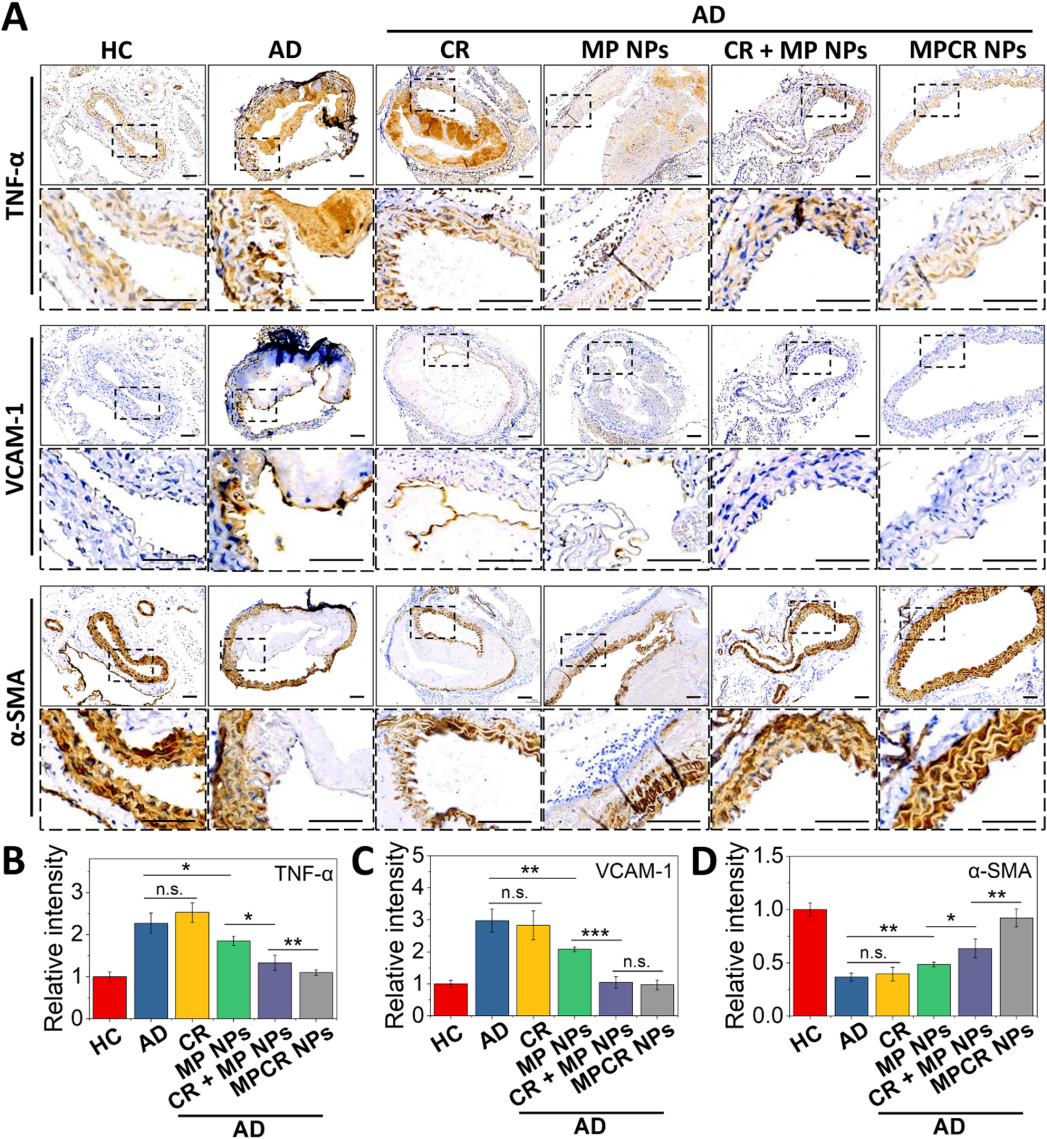
IHC staining. (**A**) IHC staining of TNF-α, VCAM-1, and α-SMA in aortic tissues. Images outlined with dashed boxes represent magnified views of selected regions. Scale bars: 100 μm. Dashed boxes indicate the regions selected for high-magnification display. All IHC stains were performed on serial sections from the same samples. Due to tissue heterogeneity and structural disruption in advanced AD lesions, adjacent sections may exhibit notable morphological differences. (**B**, **C**) Quantitative analysis (*n* = 3). n.s., non-significant difference, * *p* < 0.05, ** *p* < 0.01, *** *p* < 0.001

To further evaluate the therapeutic effects on phenotypic modulation of key vascular cell types, we additionally examined TNF-α, VCAM-1, and α-SMA expression. TNF-α is a hallmark of pro-inflammatory Mφ activation, VCAM-1 is a membrane-bound adhesion molecule induced by endothelial inflammation, and α-SMA serves as a contractile marker of differentiated VSMCs, often diminished during pathological dedifferentiation. As shown in Fig. 11, the AD group exhibited markedly increased TNF-α and VCAM-1 expression along with reduced α-SMA levels, reflecting inflammatory activation and medial degeneration. The CR + MP NPs group demonstrated greater improvement than either CR or MP NPs alone across all three markers, suggesting a synergistic effect arising from the co-delivery of RES and a dual-functional NO-donor/Gal-3 inhibitor. Notably, MPCR NPs further outperformed the mixture group, achieving greater suppression of inflammatory markers and enhanced restoration of the VSMCs contractile phenotype to near-healthy levels (Fig. 11B-D) likely attributable to the combination of their pathology-tailored Gal-3-targeting strategy (Figs. 5, 6 and 7), effective delivery characteristics (Fig. 4), and potent therapeutic effects on pathological vascular cell types (Fig. 8). This trend was consistently observed across major pathological cell types, underscoring the broad therapeutic impact of the MPCR nanoplatform. These findings further support the multi-pathway intervention capability of our system in modulating both inflammatory and structural components of AD pathology.

Recent studies explored nanotherapeutic approaches targeting AD progression. Zhou et al. reported using integrin-targeted liposome co-delivery of anti-inflammatory agents and cytokines (FNV@IL-33), administered every 3 days for 4 weeks, which reduced the AD incidence from 80% to approximately 30% [6]. Wang et al. developed NMCs, NPs coated with neutrophil membranes to enhance targeting of inflammatory sites, administered three times over 1 week, which reduced the AD incidence from 80% to 70% (which was not statistically significant) while improving survival and reducing the rupture rate [7]. Those findings underscore the challenges of effectively controlling AD progression. Compared to those approaches, our system demonstrated more-substantial efficacy, reducing the AD incidence from 87.5% to below 25% with twice-weekly dosing over 4 weeks in a similar AD model and intervention timeline. This advancement can be attributed to our platform, which leverages a pathology-tailored Gal-3 targeting strategy, where MCP not only guides localization via Gal-3 binding but also simultaneously acts as a Gal-3 inhibitor, coupled with effective delivery and controlled release of RES and NO, thereby achieving a multi-pathway intervention. Furthermore, our approach employs comparatively inexpensive materials with greater potential for clinical translation. Collectively, this study presents MPCR NPs as a promising and practical nanotherapeutic candidate for future AD treatment strategies. Notably, our work represents the first attempt to explore both a Gal-3-based targeted delivery approach and an on-demand, in situ NO-supplying therapy in the context of AD, which resulted in markedly improved therapeutic outcomes, thereby providing a strong foundation and valuable insight for advancing multi-modal nanotherapeutic research on this challenging disease.

## Conclusions

In this study, we introduce a Gal-3-targeted, multi-responsive NP platform (MPCR NPs) specifically tailored to address the complex pathophysiology of AD. By strategically co-assembling MCP, Prt, and CMCD, this nanoplatform achieved precise lesion targeting, on-demand NO release, and controlled delivery of therapeutic agents such as RES. MPCR NPs and exhibited enhanced cellular uptake across multiple AD progression-associated cell types, including ECs, Mφs, and VSMCs, effectively modulating inflammatory and degenerative processes through multimodal therapeutic synergy. Importantly, this platform demonstrated robust targeting from early through advanced disease stages, significantly enhancing the therapeutic efficacy of its constituent components in reducing the AD incidence, inflammation, vascular degeneration, and mortality. Notably, this research represents the first demonstration of NO-based nanotherapy specifically for AD, emphasizing the novel clinical potential of an early-stage intervention combined with sustained targeting efficacy throughout disease progression. Beyond AD, the versatile design of MPCR NPs offers broad applicability, given the widespread involvement of Gal-3 in various inflammatory and degenerative diseases, such as abdominal aortic aneurysms, heart failure, and pulmonary hypertension. Additionally, the platform’s capability to deliver hydrophobic therapeutics, provide gas-based therapies, and exhibit multiple stimuli-responsiveness further underscores its potential for extensive material adaptability and diverse biomedical applications.

## Experimental section

### Materials

CP (P9135, galacturonic acid ≥ 74.0%, MW 485 kDa, degree of esterification (D.e.) 57.86%) and Prt (from salmon) were purchased from Sigma-Aldrich (St. Louis, MO, USA). CMCD and polyacrylic acid (PAA, MW1.8 kDa) were also obtained from Sigma-Aldrich. RES was obtained from Tokyo Chemical Industry (TCI, Tokyo, Japan). Human umbilical vein ECs (HUVEC; BCRC No. H-UV001; RRID: CVCL_2959) were obtained from the Bioresource Collection and Research Center (BCRC; Food Industry Research and Development Institute, Hsinchu, Taiwan) and were purchased in 2023 and 2025. EA.hy926 cells (RRID: CVCL_3901, purchased in 2022), MOVAS-1 cells (RRID: CVCL_0F08, purchased in 2019), RAW264.7 cells (RRID: CVCL_0493, purchased in 2019), and THP-1 cells (RRID: CVCL_0006, purchased in 2023) were purchased from the American Type Culture Collection (ATCC, Manassas, VA, USA). All cell lines were confirmed to be free of mycoplasma and other contaminations before use. All other chemicals and reagents were of analytical grade and were used as received.

### MCP preparation

CP (7.5 g) was dissolved in 500 mL of ultrapure water and stirred at room temperature until fully solubilized. The pH was adjusted to 10 by the dropwise addition of 10 M NaOH, forming a clear brown solution. The mixture was stirred at 55 °C for 1 h, cooled to room temperature, and adjusted to pH 3.0 using 3 M HCl. After stirring for 24 h, the solution had become a turbid suspension. The pH was then readjusted to 6.8 with 3 M NaOH. MCP was precipitated by adding 95% ethanol (1:4 v/v), allowed to stand for 10 min, and collected by centrifugation at 10,000 rpm for 10 min at 20 °C. The pellet was washed with acetone (1:4 v/v), mechanically dispersed, and centrifuged again under the same conditions. The final product was dried at 37 °C and stored at 4 °C [30]. The molecular weight of MCP was determined by gel permeation chromatography (GPC) using a Shimadzu LC-20 Prominence system (Kyoto, Japan) equipped with a refractive index detector and a TSKgel G4000PWxL column (Tokyo, Japan). Ultrapure water served as the mobile phase (0.5 mL/min). Filtered MCP samples (10 mg/mL, 5 µL injection) were analyzed using a dextran standard kit (Agilent Technologies, CA, USA) for calibration. The D.e. was determined by ^1^H NMR spectroscopy using an Agilent 600 MHz NMR spectrometer. MCP was dissolved in D_2_O, and D.e. was calculated by integrating the methyl ester proton signal (~4.3 ppm) relative to the anomeric proton signals of galacturonic acid residues (~ 4.9–5.2 ppm).

### CR preparation

The CR complex was prepared to enhance the aqueous solubility and NP compatibility of RES. Briefly, CMCD (500 mg) was dissolved in 45 mL of ultrapure water, and RES (50 mg) was dissolved in 5 mL of ethanol. The two solutions were mixed and stirred at 800 rpm for 24 h at room temperature. The resulting mixture was filtered through a 0.45-µm membrane to remove uncomplexed RES, and the filtrate was lyophilized to yield CR powder. The product was characterized by Fourier transform infrared (FTIR) spectroscopy (FTS-155, Digilab, MA, USA), ^1^H NMR (Agilent 600 MHz) and fluorescence-based quantification of RES loading used a microplate spectrophotometer (Tecan Spark, Männedorf, Switzerland) at Ex/Em wavelengths of 310/380 nm.

### MPCR NPs preparation

Self-assembled NPs were prepared via polyelectrolyte complexation. MCP (4 mg/mL), Prt (4 mg/mL), and CMCD (20 mg/mL) were separately dissolved in PBS and mixed under constant stirring at various weight ratios to determine the optimal formulation. The MP NPs were obtained at a MCP: Prt weight ratio of 1:0.66. For MPC NPs, CMCD was first pre-mixed with MCP prior to the addition of Prt, yielding a final MCP: Prt: CMCD weight ratio of 1:0.67:6.67. For drug-loaded MPCR NPs, CMCD was replaced with an equivalent weight of CR. The hydrodynamic size and ζ-potential of the NPs were measured by dynamic light scattering (ZEN3600, Malvern Instruments, Worcestershire, UK). The morphology was visualized by transmission electron microscopy (TEM; H-7700, Hitachi, Tokyo, Japan). The secondary structure of Prt in different formulations was analyzed using circular dichroism (CD) spectroscopy (AVIV model 400, Aviv Biomedical, Lakewood, NJ, USA), and structural deconvolution was performed using the BeStSel™ method [71].

### Anti-autoxidation and hydroxyl radical scavenging

The measurement was performed at a resonance frequency of 9.42 GHz with a microwave power of 6.33 mW, a modulation frequency of 100 kHz, and a modulation amplitude of 1.0 G. The anti-autoxidation property of MPCR NPs was evaluated by monitoring the UV–vis spectrum of RES in PBS/DMSO buffer. Samples were prepared at a final RES-equivalent concentration of 16 µM, incubated at 37 °C, and analyzed using a microplate spectrophotometer (Tecan Spark, Männedorf, Switzerland). The hydroxyl radical (•OH)-scavenging activity was evaluated by electron spin resonance (ESR) spectroscopy using 5,5-dimethyl-1-pyrroline N-oxide (DMPO, 100 mM) as a spin-trapping agent. Samples were mixed with H_2_O_2_ (1 mM), FeSO_4_ (2 mM), and DMPO, immediately transferred to capillary tubes, and analyzed using an EMX 10/12 ESR spectrometer (Bruker EMXmicro, MA, USA). Quantification was based on the relative decrease in the ESR signal intensity. The scavenging rate (%) was calculated using the following equation:

$$\:\mathrm{S}\mathrm{c}\mathrm{a}\mathrm{v}\mathrm{e}\mathrm{n}\mathrm{g}\mathrm{i}\mathrm{n}\mathrm{g}\:\mathrm{r}\mathrm{a}\mathrm{t}\mathrm{e}\:\left(\mathrm{\%}\right)=\left(1-\:\frac{{I}_{sample}}{{I}_{control}}\right)\times\:100$$

where *I*_sample_ and *I*_control_ represent the peak intensity of the DMPO–OH adduct in the presence and absence of the test sample, respectively.

### Colloidal stability, encapsulation efficiency, and drug release

The colloidal stability of MPCR NPs was evaluated in PBS (pH 7.4) and DMEM supplemented with 10% FBS at 37 °C for up to 72 h. The hydrodynamic diameter and PDI were recorded at designated time points to assess the NP stability. The encapsulation efficiency (EE%) of RES in MPCR NPs was determined by quantifying unencapsulated CR in the supernatant after centrifugation at 12,000 rpm for 10 min. The fluorescence intensity of RES was measured using a spectrophotometer (Tecan Spark, Männedorf, Switzerland). The EE (%) was calculated using the following equation:

$$\:\mathrm{E}\mathrm{E}\:\left(\mathrm{\%}\right)=\left(\:\frac{{W}_{total}-\:{W}_{free}}{{W}_{total}}\right)\times\:100$$

where *W*_total_ is the amount of CR initially added, and *W*_free_ is the amount detected in the supernatant.

Cumulative CR release from MPCR NPs was assessed using the dialysis bag method (CelluSep H1, MFPI, USA). Samples were dialyzed against a 9-fold volume of PBS (pH 7.4, 6.8, or 5.0), with or without trypsin (2,500 USP units/mg Prt), at 37 °C under gentle shaking. At predetermined intervals, aliquots of the external medium were collected and replaced with fresh buffer. Released RES was quantified by fluorescence spectroscopy (Ex/Em = 310/380 nm), and the cumulative release was calculated using the following equation:

$$\:\mathrm{C}\mathrm{u}\mathrm{m}\mathrm{u}\mathrm{l}\mathrm{a}\mathrm{t}\mathrm{i}\mathrm{v}\mathrm{e}\:\mathrm{r}\mathrm{e}\mathrm{l}\mathrm{e}\mathrm{a}\mathrm{s}\mathrm{e}\:\left(\mathrm{\%}\right)=\left(\:\frac{{P}_{t}-\:{P}_{(t-1)}}{{W}_{encapsulated}}\right)\times\:100$$

where *P*_t_ is the amount of RES released at time *t*, and *P*_(t−1)_ is the cumulative release at previous time points.

NO release from MPCR NPs and free Prt, at an equivalent concentration of 0.3 mg/mL, were evaluated using the Griess reagent. Samples were incubated with H_2_O_2_ (100 µM), with or without trypsin, and nitrite levels were determined by the absorbance at 540 nm.

### Cell culture and treatments

All cells were cultured in their respective media supplemented with 10% FBS and 1% penicillin-streptomycin, and maintained at 37 °C in a humidified incubator with 5% CO_2_. To model vascular inflammation, HUVECs and MOVAS cells were treated with PMA (100 nM) for 24 h to induce a pro-inflammatory phenotype. RAW264.7 cells were stimulated with LPS (100 ng/mL) and IFN-γ (20 ng/mL) for 24 h to mimic an inflammatory microenvironment. Cell viability was assessed using the Cell Counting Kit-8 (CCK-8; Abbkine, Redlands, CA, USA) according to the manufacturer’s protocol. After 2 h incubation with the CCK-8 reagent, absorbance was measured at 450 nm using a microplate reader (Tecan Spark, Männedorf, Switzerland). Extracellular NO levels were assessed using the Griess assay. Intracellular NO levels were determined using DAF-2DA (10 µM, 30 min) and visualized through CLSM (Leica Stellaris 8 system, Leica Microsystems, Wetzlar, Germany). Intracellular reactive oxygen species (ROS) generation was evaluated using 2′,7′-dichlorodihydrofluorescein diacetate (DCFH-DA, 5 µM; Invitrogen, USA). Cells were incubated with DCFH-DA for 30 min at 37 °C, washed with PBS, and the fluorescence was measured (Ex/Em = 488/515 nm).

### Cellular uptake

Cells were stimulated with proinflammatory agents (PMA + LPS + IFN-γ for THP-1 and PMA for HUVECs and MOVAS cells) for 24 h to upregulate Gal-3. The expression of Gal-3 was confirmed by CLSM. Fluorescence (FL)-labeled NPs were incubated with cells for 2 h, and uptake was quantified by CLSM or flow cytometry. Competitive inhibition assays using free MCP (as the Gal-3 blocking agent), PAA-substituted NPs (as the non-targeting control), or Gal-3 siRNA knockdown were performed to assess targeting specificity.

### Western blotting

Proteins were extracted from cells using RIPA buffer plus protease inhibitors. SDS-PAGE was used to separate an equal amount of protein, which was then transferred to the PVDF membrane. After blocking, membranes were incubated with diluted primary antibodies to detect iNOS (1:2000, Abcam, Cat# ab178945), NLRP3 (1:1000, Cell signaling, Cat# 15101 s), COX-2 (1:000, proteintech, Cat# 12375-1-AP), α-SMA (1:1000, Sigma-Aldrch, Cat# A5228), SM22α (1:1000, Abcam, Cat# ab14106), vimentin (1:1000, Abcam, Cat# ab92547), OPN (1:1000, Proteintech, Cat #22952-1-AP) and overnight at 4 °C followed by incubation with a HRP-conjugated secondary antibody (Jackson Immuno Research, West Grove, PA, USA). Proteins were visualized using an enhance chemiluminescence (ECL) detection substrate (Millipore, Billerica, MA, USA) on UVP. β-actin and GAPDH were detected as a loading control.

### In vivo AD model, targeting, and validation

All experimental designs and procedures were approved by the Institutional Animal Care and Use Committee (IACUC) of Taipei Medical University (LAC-2021-0464). Animals were housed and cared for in accordance with ARRIVE guidelines at an accredited facility. Three-week-old male C57BL/6 mice were administered a solution of BAPN (Sigma- Aldrich, St. Louis, MO, USA), which was dissolved in drinking water at a concentration of 1 g/kg per day for 4 weeks (*n* = 8). After 4 weeks, the mice were implanted dorsally with a subcutaneous osmotic mini-pump (Model 1003D Micro-osmotic Pump; Alzet, Cupertino, California, USA) to infuse angiotensin II (Ang-II) at a concentration of 1 µg/kg/min. Each treatment was administered via tail vein injection at 200 µL per dose, twice weekly for 3 weeks (approximate RES dose of 2.58 mg/kg per injection). The MPCR NP formulation used for in vivo administration contained MCP (1.3 mg/mL), Prt (0.9 mg/mL), and CR (8.5 mg/mL), corresponding to a RES-equivalent concentration of 322.6 µg/mL. All comparator groups (CR, MP NPs, CR + MP NPs) were administered at equivalent doses of each functional component based on their concentrations in the MPCR NP formulation, using the same injection volume and frequency. Cy7-labeled MPCR NPs were administered intravenously, and in vivo fluorescence imaging was performed using PerkinElmer IVIS Lumina XRMS Series III Imaging System (Waltham, USA) with a Cy7 filter at 6 h post-injection, following imaging procedures similar to previous vascular-targeting nanoprobe studies [72]. Aortic tissue was excised for ex vivo imaging, fluorescence quantification, and cryosection analysis.

### Toxicological evaluation

At week 4, major organs were collected for H&E staining to assess histopathology. Serum biochemical markers (SGOT, SGPT, ALKP, BUN, LDH, CRE) were measured using an automated analyzer to evaluate systemic toxicity. Differences between AD and MPCR NP groups were compared to assess safety and protective effects.

### Pharmacokinetic analysis

Pharmacokinetic evaluation was conducted in AD mice established as described above. BALB/c mice were intravenously administered with CR or MPCR NPs at a RES-equivalent dose of 2.58 mg/kg. Blood samples were collected at predetermined time points, and plasma was isolated by centrifugation and stored at − 80 °C until analysis. For LC-MS/MS quantification, 30 µL of plasma was mixed with 200 µL of methanol/acetonitrile (1:1, v/v) containing 0.1% ascorbic acid and 100 ng/mL resveratrol-d_4_ (MedChemExpress, NJ, USA) as an internal standard. After centrifugation (14,000 g, 10 min, 4 °C), the supernatant was filtered through a 0.22 μm PVDF membrane and subjected to analysis. Chromatographic separation was performed using a Kinetex C18 column (100 × 4.6 mm, 2.6 μm, 100 Å) on an Agilent 1290 Infinity II LC system (Waldbronn, Germany) with a gradient elution of 0.1% formic acid in water (A) and acetonitrile (B) at a flow rate of 0.3 mL/min. An Agilent 6470 triple quadrupole mass spectrometer (Waldbronn, Germany) equipped with an ESI source operating in negative ion mode was used for detection. Multiple reaction monitoring transitions were m/z 227→185 for RES and m/z 231→189 for resveratrol-d_4_. The assay demonstrated good linearity over the range of 5–1000 ng/mL, with a lower limit of quantification of 5 ng/mL.

### Histological analysis and IHC staining

After perfusion fixation, approximately 4–5 mm segments of the ascending aorta (from the aortic root to just before the brachiocephalic artery) and proximal descending thoracic aorta (just above the diaphragm) were excised. Tissues were fixed in 4% formaldehyde overnight, dehydrated, embedded in paraffin, and sectioned into 4 μm slices. These sections were stained using hematoxylin and eosin (H&E) and Verhoeff-van Gieson (VVG) staining techniques and examined under a light microscope. For immunohistochemistry, the sections were deparaffinized into water for antigen retrieval and incubated in 3% hydrogen peroxidase (H_2_O_2_). After blocking with 5% BSA, the sections were incubated with primary antibodies against Gal-3 (1:50; GeneTex, Cambridge, MA, USA, Cat# GTX125897), CD68 (1:200; Abcam, Cat# ab283654), MMP-9 (1:200; Abcam, Cat# ab228402), a-SMA (1:1000 abcam #ab5694), TNF-a (1:200 iREAL #IR106-P2), and VCAM (1:300 abcam #ab134047) at 4 °C overnight and against secondary antibodies for 30 min at room temperature. Then, a diaminobenzidine horseradish peroxidase system was used. Finally, the sections were counterstained with hematoxylin and viewed under a light microscope.

### Statistical analysis

Results are presented as the mean ± standard deviation (*n* = 3–6). Statistical analysis was performed using Student’s *t*-test. *p* values of < 0.05 (* *p* < 0.05), < 0.01 (** *p* < 0.01), and < 0.001 (*** *p* < 0.001) were considered statistically significant. All statistical analyses were conducted using SPSS vers. 22.0 (IBM, Armonk, NY, USA). Survival curve analysis (Fig. 9D) was performed using the Kaplan–Meier method with log-rank (Mantel–Cox) tests to compare survival distributions between groups. Aortic rupture incidence (Fig. 9E) was evaluated using Fisher’s exact test. These analyses were performed using GraphPad Prism version 9.0.0 (San Diego, CA, USA).

## Supplementary Information

Supplementary Material 1

## Data Availability

Data is provided within the manuscript or supplementary information files.

## References

[1] Evangelista A, Isselbacher EM, Bossone E, Gleason TG, Eusanio MD, Sechtem U, et al. Insights from the international registry of acute aortic dissection: a 20-year experience of collaborative clinical research. Circulation. 2018;137:1846–60. 10.1161/CIRCULATIONAHA.117.031264. 29685932 10.1161/CIRCULATIONAHA.117.031264

[2] Nienaber CA, Eagle KA. Aortic dissection: new frontiers in diagnosis and management: part II: therapeutic management and follow-up. Circulation. 2003;108:772–8. 10.1161/01.CIR.0000087400.48663.19. 12912795 10.1161/01.CIR.0000087400.48663.19

[3] Nienaber CA, Rousseau H, Eggebrecht H, Kische S, Fattori R, Rehders TC, et al. Randomized comparison of strategies for type B aortic dissection: the INvestigation of STEnt grafts in Aortic Dissection (INSTEAD) trial. Circulation. 2009;120:2519–28. 10.1161/CIRCULATIONAHA.109.886408. 19996018 10.1161/CIRCULATIONAHA.109.886408

[4] Kurihara T, Shimizu-Hirota R, Shimoda M, Adachi T, Shimizu H, Weiss SJ, et al. Neutrophil-derived matrix metalloproteinase 9 triggers acute aortic dissection. Circulation. 2012;126:3070–80. 10.1161/CIRCULATIONAHA.112.097097. 23136157 10.1161/CIRCULATIONAHA.112.097097

[5] Chen Y, He Y, Wei X, Jiang D-S. Targeting regulated cell death in aortic aneurysm and dissection therapy. Pharmacol Res. 2022;176:106048. 10.1016/j.phrs.2021.106048. 34968685 10.1016/j.phrs.2021.106048

[6] Ji C, Wang X, Xue B, Li S, Li J, Qiao B, et al. A fluorescent nano vector for early diagnosis and enhanced Interleukin-33 therapy of thoracic aortic dissection. Biomaterials. 2023;293:121958. 10.1016/j.biomaterials.2022.121958. 36566550 10.1016/j.biomaterials.2022.121958

[7] Li T-x, Yang Y-y, Zong J-b, Li M, Fu X-x, Jiang X-x, et al. Activated neutrophil membrane-coated tRF-Gly-CCC nanoparticles for the treatment of aortic dissection/aneurysm. J Control Release. 2025;378:334–49. 10.1016/j.jconrel.2024.12.015. 39672274 10.1016/j.jconrel.2024.12.015

[8] Xu L, Burke A. Acute medial dissection of the ascending aorta: evolution of reactive histologic changes. Am J Surg Pathol. 2013;37:1275–82. 10.1097/PAS.0b013e318294adc3. 23774175 10.1097/PAS.0b013e318294adc3

[9] Toczek J, Meadows JL, Sadeghi MM. Novel molecular imaging approaches to abdominal aortic aneurysm risk stratification. Circ Cardiovasc Imaging. 2016;9:e003023. 10.1161/CIRCIMAGING.115.003023. 26763279 10.1161/CIRCIMAGING.115.003023 PMC4714781

[10] Oroojalian F, Beygi M, Baradaran B, Mokhtarzadeh A, Shahbazi MA. Immune cell membrane-coated biomimetic nanoparticles for targeted cancer therapy. Small. 2021;17:2006484. 10.1002/smll.202006484. 10.1002/smll.202006484 33577127

[11] Wang D, Wang S, Zhou Z, Bai D, Zhang Q, Ai X, et al. White blood cell membrane-coated nanoparticles: recent development and medical applications. Adv Healthc Mater. 2022;11:2101349. 10.1002/adhm.202101349. 10.1002/adhm.202101349 34468090

[12] Papaspyridonos M, McNeill E, de Bono JP, Smith A, Burnand KG, Channon KM, et al. Galectin-3 is an amplifier of inflammation in atherosclerotic plaque progression through macrophage activation and monocyte chemoattraction. Arterioscler Thromb Vasc Biol. 2008;28:433–40. 10.1161/ATVBAHA.107.159160. 18096829 10.1161/ATVBAHA.107.159160

[13] Lu H-Y, Shih C-M, Huang C-Y, Wu AT, Cheng T-M, Mi F-L, et al. Galectin-3 modulates macrophage activation and contributes smooth muscle cells apoptosis in abdominal aortic aneurysm pathogenesis. Int J Mol Sci. 2020;21:8257. 10.3390/ijms21218257. 33158139 10.3390/ijms21218257 PMC7663490

[14] Li T, Zha L, Luo H, Li S, Zhao L, He J, et al. Galectin-3 mediates endothelial-to-mesenchymal transition in pulmonary arterial hypertension. Aging Dis. 2019;10:731. 10.14336/AD.2018.1001. 31440380 10.14336/AD.2018.1001 PMC6675525

[15] Blanda V, Bracale UM, Di Taranto MD, Fortunato G. Galectin-3 in cardiovascular diseases. Int J Mol Sci. 2020;21:9232. 10.3390/ijms21239232. 33287402 10.3390/ijms21239232 PMC7731136

[16] Chen X, Lin J, Hu T, Ren Z, Li L, Hameed I, et al. Galectin-3 exacerbates ox‐LDL‐mediated endothelial injury by inducing inflammation via integrin β1‐RhoA‐JNK signaling activation. J Cell Physiol. 2019;234:10990–1000. 10.1002/jcp.27910. 30536538 10.1002/jcp.27910 PMC6590151

[17] Tieu BC, Lee C, Sun H, LeJeune W, Recinos A, Ju X, et al. An adventitial IL-6/MCP1 amplification loop accelerates macrophage-mediated vascular inflammation leading to aortic dissection in mice. J Clin Invest. 2009;119:3637–51. 10.1172/JCI38308. 19920349 10.1172/JCI38308 PMC2786788

[18] Menini S, Iacobini C, Ricci C, Blasetti Fantauzzi C, Salvi L, Pesce CM, et al. The galectin-3/RAGE dyad modulates vascular osteogenesis in atherosclerosis. Cardiovasc Res. 2013;100:472–80. 10.1093/cvr/cvt206. 23975852 10.1093/cvr/cvt206

[19] Ganizada BH, Veltrop RJ, Akbulut AC, Koenen RR, Accord R, Lorusso R, et al. Unveiling cellular and molecular aspects of ascending thoracic aortic aneurysms and dissections. Basic Res Cardiol. 2024;119:371–95. 10.1007/s00395-024-01053-1. 38700707 10.1007/s00395-024-01053-1 PMC11143007

[20] Calvier L, Miana M, Reboul P, Cachofeiro V, Martinez-Martinez E, De Boer RA, et al. Galectin-3 mediates aldosterone-induced vascular fibrosis. Arterioscler Thromb Vasc Biol. 2013;33:67–75. 10.1161/ATVBAHA.112.300569. 23117656 10.1161/ATVBAHA.112.300569

[21] Martínez-Martínez E, López-Ándres N, Jurado-López R, Rousseau E, Bartolomé MV, Fernández-Celis A, et al. Galectin-3 participates in cardiovascular remodeling associated with obesity. Hypertension. 2015;66:961–9. 10.1161/HYPERTENSIONAHA.115.06032. 26351031 10.1161/HYPERTENSIONAHA.115.06032

[22] Vergaro G, Prud’Homme M, Fazal L, Merval R, Passino C, Emdin M, et al. Inhibition of galectin-3 pathway prevents isoproterenol-induced left ventricular dysfunction and fibrosis in mice. Hypertension. 2016;67:606–12. 10.1161/HYPERTENSIONAHA.115.06161. 26781273 10.1161/HYPERTENSIONAHA.115.06161

[23] Dobiasch S, Szanyi S, Kjaev A, Werner J, Strauss A, Weis C, et al. Synthesis and functionalization of protease-activated nanoparticles with tissue plasminogen activator peptides as targeting moiety and diagnostic tool for pancreatic cancer. J Nanobiotechnology. 2016;14:1–18. 10.1186/s12951-016-0236-3. 27993133 10.1186/s12951-016-0236-3 PMC5168863

[24] Ma X, Li X, Shi J, Yao M, Zhang X, Hou R, et al. Host–guest polypyrrole nanocomplex for three-stimuli‐responsive drug delivery and imaging‐guided chemo‐photothermal synergetic therapy of refractory thyroid cancer. Adv Healthc Mater. 2019;8:1900661. 10.1002/adhm.201900661. 10.1002/adhm.201900661 31389191

[25] Balakrishnan B, Subramanian S, Mallia MB, Repaka K, Kaur S, Chandan R, et al. Multifunctional core–shell glyconanoparticles for galectin-3-targeted, trigger-responsive combination chemotherapy. Biomacromolecules. 2020;21:2645–60. 10.1021/acs.biomac.0c00358. 32484667 10.1021/acs.biomac.0c00358

[26] Bai S, Sun Y, Cheng Y, Ye W, Jiang C, Liu M, et al. MCP mediated active targeting calcium phosphate hybrid nanoparticles for the treatment of orthotopic drug-resistant colon cancer. J Nanobiotechnology. 2021;19:1–20. 10.1186/s12951-021-01115-9. 34789268 10.1186/s12951-021-01115-9 PMC8600743

[27] Buaron N, Mangraviti A, Volpin F, Liu A, Pedone M, Sankey E, et al. Pectic galactan polysaccharide-based gene delivery system for targeting neuroinflammation. Adv Funct Mater. 2021;31:2100643. 10.1002/adfm.202100643.

[28] Perera KD, Ghumman M, Sorkhdini P, Norbrun C, Negash S, Zhou Y, et al. Citrus pectin-coated inhalable PLGA nanoparticles for treatment of pulmonary fibrosis. J Mater Chem B. 2025;13:3325–39. 10.1039/D4TB01682C. 39918485 10.1039/d4tb01682c PMC11804936

[29] Wu D, Zheng J, Hu W, Zheng X, He Q, Linhardt RJ, et al. Structure-activity relationship of citrus segment membrane RG-I pectin against Galectin-3: the galactan is not the only important factor. Carbohydr Polym. 2020;245:116526. 10.1016/j.carbpol.2020.116526. 32718630 10.1016/j.carbpol.2020.116526

[30] Lin C, Mi F-L, Cha C-Y, Hsu F-Y, Ayu Ulfadillah S, Tsai M-L, et al. Structurally reprogrammed modified citrus pectin (MCP) enables potentiated galectin-3 sequestration and injectable carboxymethyl chitosan/berberine hydrogel construction for osteoarthritis immunotherapy. Mater Today Bio. 2025;102330. 10.1016/j.mtbio.2025.102330. 41080725 10.1016/j.mtbio.2025.102330 PMC12509201

[31] Eliaz I, Raz A. Pleiotropic effects of modified citrus pectin. Nutrients. 2019;11:2619. 10.3390/nu11112619. 31683865 10.3390/nu11112619 PMC6893732

[32] Li J, Zhang J, Yu P, Xu H, Wang M, Chen Z, et al. ROS-responsive & scavenging NO nanomedicine for vascular diseases treatment by inhibiting endoplasmic reticulum stress and improving NO bioavailability. Bioact Mater. 2024;37:239–52. 10.1016/j.bioactmat.2024.03.010. 38549770 10.1016/j.bioactmat.2024.03.010 PMC10973783

[33] Lin C, Cheng T-M, Liu Y-C, Hsu F-Y, Shih C-M, Tsai M-L, et al. Dual-targeting EGCG/NO-supplying protein assembled nanoparticles with multi-synergistic effects against atherosclerosis. Chem Eng J. 2024;493:152755. 10.1016/j.cej.2024.152755.

[34] Yang Z, Yang Y, Xiong K, Li X, Qi P, Tu Q, et al. Nitric oxide producing coating mimicking endothelium function for multifunctional vascular stents. Biomaterials. 2015;63:80–92. 10.1016/j.biomaterials.2015.06.016. 26093790 10.1016/j.biomaterials.2015.06.016

[35] Oh Y, Jeong H, Lim S, Hong J. Controlled nitric oxide release using poly (lactic-co-glycolic acid) nanoparticles for anti-inflammatory effects. Biomacromolecules. 2020;21:4972–9. 10.1021/acs.biomac.0c01176. 33147008 10.1021/acs.biomac.0c01176

[36] Bonetti J, Corti A, Lerouge L, Pompella A, Gaucher C. Phenotypic modulation of macrophages and vascular smooth muscle cells in atherosclerosis—nitro-redox interconnections. Antioxidants. 2021;10:516. 10.3390/antiox10040516. 33810295 10.3390/antiox10040516 PMC8066740

[37] Ma G-G, Hao G-W, Lai H, Yang X-M, Liu L, Wang C-S, et al. Initial clinical impact of inhaled nitric oxide therapy for refractory hypoxemia following type A acute aortic dissection surgery. J Thorac Dis. 2019;11:495. 10.21037/jtd.2019.01.42. 30962993 10.21037/jtd.2019.01.42 PMC6409278

[38] Zhang H, Liu Y, Meng X, Yang D, Shi S, Liu J, et al. Effects of inhaled nitric oxide for postoperative hypoxemia in acute type A aortic dissection: a retrospective observational study. J Cardiothorac Surg. 2020;15:1–9. 10.1186/s13019-020-1069-6. 31969173 10.1186/s13019-020-1069-6 PMC6977331

[39] Wang K, Zhao J, Zhang W, Zhu M, Xu M, Li D, et al. Resveratrol attenuates aortic dissection by increasing endothelial barrier function through the SIRT1 pathway. J Cardiovasc Pharmacol. 2020;76:86–93. 10.1097/FJC.0000000000000837. 32324654 10.1097/FJC.0000000000000837 PMC7340227

[40] Tao Y, Li G, Yang Y, Wang Z, Wang S, Li X, et al. Epigenomics in aortic dissection: from mechanism to therapeutics. Life Sci. 2023;335:122249. 10.1016/j.lfs.2023.122249. 37940070 10.1016/j.lfs.2023.122249

[41] Bosshardt D, Van Andel M, Schrauben E, Gottwald L, Van Kimmenade R, Scholte A et al. The effect of resveratrol on aortic growth and function in patients with marfan syndrome. European Heart Journal. 2023;44:ehad655. 2033. 10.1136/heartjnl-2024-324343

[42] Hibender S, Franken R, Van Roomen C, Ter Braake A, Van Der Made I, Schermer EE, et al. Resveratrol inhibits aortic root dilatation in the Fbn1C1039G/+ Marfan mouse model. Arterioscler Thromb Vasc Biol. 2016;36:1618–26. 10.1161/ATVBAHA.116.307841. 27283746 10.1161/ATVBAHA.116.307841 PMC4961273

[43] Ren B, Kwah MX-Y, Liu C, Ma Z, Shanmugam MK, Ding L, et al. Resveratrol for cancer therapy: challenges and future perspectives. Cancer Lett. 2021;515:63–72. 10.1016/j.canlet.2021.05.001. 34052324 10.1016/j.canlet.2021.05.001

[44] Amri A, Chaumeil J, Sfar S, Charrueau C. Administration of resveratrol: what formulation solutions to bioavailability limitations? J Control Release. 2012;158:182–93. 10.1016/j.jconrel.2011.09.083. 21978644 10.1016/j.jconrel.2011.09.083

[45] Berman AY, Motechin RA, Wiesenfeld MY, Holz MK. The therapeutic potential of resveratrol: a review of clinical trials. NPJ Precis Oncol. 2017;1:35. 10.1038/s41698-017-0038-6. 28989978 10.1038/s41698-017-0038-6 PMC5630227

[46] Ong CS, Nam L, Yesantharao P, Dong J, Canner JK, Teuben RJ, et al. The strongest risk factor for operative mortality in acute type A aortic dissection is acidosis: validation of risk model. Semin Thorac Cardiovasc Surg. 2020. 10.1053/j.semtcvs.2020.02.023. 32105786 10.1053/j.semtcvs.2020.02.023

[47] Pincemail J, Tchana-Sato V, Courtois A, Musumeci L, Cheramy-Bien J-P, Munten J, et al. Alteration of blood oxidative stress status in patients with thoracic aortic dissection: a pilot study. Antioxidants. 2023;12:1106. 10.3390/antiox12051106. 37237972 10.3390/antiox12051106 PMC10215099

[48] Wang X, Parvathaneni V, Shukla SK, Kulkarni NS, Muth A, Kunda NK, et al. Inhalable resveratrol-cyclodextrin complex loaded biodegradable nanoparticles for enhanced efficacy against non-small cell lung cancer. Int J Biol Macromol. 2020;164:638–50. 10.1016/j.ijbiomac.2020.07.124. 32693132 10.1016/j.ijbiomac.2020.07.124

[49] Felber AE, Dufresne M-H, Leroux J-C. pH-sensitive vesicles, polymeric micelles, and nanospheres prepared with polycarboxylates. Adv Drug Deliv Rev. 2012;64:979–92. 10.1016/j.addr.2011.09.006. 21996056 10.1016/j.addr.2011.09.006

[50] Fernandes A, Ivanova G, Brás NF, Mateus N, Ramos MJ, Rangel M, et al. Structural characterization of inclusion complexes between cyanidin-3-O-glucoside and β-cyclodextrin. Carbohydr Polym. 2014;102:269–77. 24507282 10.1016/j.carbpol.2013.11.037

[51] Hadadian M, Allahyari R, Mahdavi B, Rezaei-Seresht E. Design, characterization, and in vitro evaluation of magnetic carboxymethylated β-cyclodextrin as a pH-sensitive carrier system for amantadine delivery: a novel approach for targeted drug delivery. RSC Adv. 2025;15:446–59. 10.1039/D4RA06269H. 39758925 10.1039/d4ra06269h PMC11698272

[52] Wang W, Feng Y, Chen W, Adie K, Liu D, Yin Y. Citrus pectin modified by microfluidization and ultrasonication: improved emulsifying and encapsulation properties. Ultrason Sonochem. 2021;70:105322. 10.1016/j.ultsonch.2020.105322. 32906066 10.1016/j.ultsonch.2020.105322 PMC7786527

[53] Hammoud Z, Khreich N, Auezova L, Fourmentin S, Elaissari A, Greige-Gerges H. Cyclodextrin-membrane interaction in drug delivery and membrane structure maintenance. Int J Pharm. 2019;564:59–76. 10.1016/j.ijpharm.2019.03.063. 30959238 10.1016/j.ijpharm.2019.03.063

[54] Zhang J, Ma PX. Cyclodextrin-based supramolecular systems for drug delivery: recent progress and future perspective. Adv Drug Deliv Rev. 2013;65:1215–33. 10.1016/j.addr.2013.05.001. 23673149 10.1016/j.addr.2013.05.001 PMC3885994

[55] Liu C-L, Liu X, Wang Y, Deng Z, Liu T, Sukhova GK, et al. Reduced Nhe1 (Na+-H + Exchanger-1) function protects ApoE-deficient mice from Ang II (Angiotensin II)–induced abdominal aortic aneurysms. Hypertension. 2020;76:87–100. 10.1161/HYPERTENSIONAHA.119.14485. 32475310 10.1161/HYPERTENSIONAHA.119.14485 PMC7289683

[56] Liao M, Liu Z, Bao J, Zhao Z, Hu J, Feng X, et al. A proteomic study of the aortic media in human thoracic aortic dissection: implication for oxidative stress. J Thorac Cardiovasc Surg. 2008;136:65–72. 10.1016/j.jtcvs.2007.11.017. 18603055 10.1016/j.jtcvs.2007.11.017

[57] Porras AM, Westlund JA, Evans AD, Masters KS. Creation of disease-inspired biomaterial environments to mimic pathological events in early calcific aortic valve disease. Proc Natl Acad Sci U S A. 2018;115:E363-71. 10.1073/pnas.1704637115. 29282325 10.1073/pnas.1704637115 PMC5776956

[58] Kumar R, Roy I, Ohulchanskky TY, Vathy LA, Bergey EJ, Sajjad M, et al. *In Vivo* biodistribution and clearance studies using multimodal organically modified silica nanoparticles. ACS Nano. 2010;4:699–708. 10.1021/nn901146y. 20088598 10.1021/nn901146y PMC2827663

[59] Pei C, Wang X, Lin Y, Fang L, Meng S. Inhibition of galectin-3 alleviates cigarette smoke extract‐induced autophagy and dysfunction in endothelial progenitor cells. Oxid Med Cell Longev. 2019;2019:7252943. 10.1155/2019/7252943. 31737173 10.1155/2019/7252943 PMC6815545

[60] Parsamanesh N, Asghari A, Sardari S, Tasbandi A, Jamialahmadi T, Xu S, et al. Resveratrol and endothelial function: a literature review. Pharmacol Res. 2021;170:105725. 10.1016/j.phrs.2021.105725. 34119624 10.1016/j.phrs.2021.105725

[61] Wang X, Zhang H, Cao L, He Y, Ma A, Guo W. The role of macrophages in aortic dissection. Front Physiol. 2020;11:54. 10.3389/fphys.2020.00054. 32116765 10.3389/fphys.2020.00054 PMC7013038

[62] Pan X, Wang H, Zheng Z, Huang X, Yang L, Liu J, et al. Pectic polysaccharide from *Smilax china* L. ameliorated ulcerative colitis by inhibiting the galectin-3/NLRP3 inflammasome pathway. Carbohydr Polym. 2022;277:118864. 10.1016/j.carbpol.2021.118864. 34893269 10.1016/j.carbpol.2021.118864

[63] Wang Z, Zhang S, Xiao Y, Zhang W, Wu S, Qin T et al. NLRP3 inflammasome and inflammatory diseases. Oxidative medicine and cellular longevity. 2020;2020:4063562. 10.3389/fphys.2020.00054 10.1155/2020/4063562 PMC7049400 32148650

[64] Liu R, Bauer AJ, Martin KA. A new editor of smooth muscle phenotype. Volume 119. Hagerstown, MD: Lippincott Williams & Wilkins; 2016. pp. 401–3. 10.1161/CIRCRESAHA.116.309218 PMC4966897 27458192

[65] Allahverdian S, Chaabane C, Boukais K, Francis GA, Bochaton-Piallat M-L. Smooth muscle cell fate and plasticity in atherosclerosis. Cardiovasc Res. 2018;114:540–50. 10.1093/cvr/cvy022. 29385543 10.1093/cvr/cvy022 PMC5852505

[66] Mu S, Guo S, Wang X, Zhan Y, Li Y, Jiang Y, et al. Effects of deferoxamine on the osteogenic differentiation of human periodontal ligament cells. Mol Med Rep. 2017;16:9579–86. 10.3892/mmr.2017.7810. 29039615 10.3892/mmr.2017.7810

[67] Hartmann F, Gorski DJ, Newman AA, Homann S, Petz A, Owsiany KM, et al. SMC-Derived Hyaluronan Modulates Vascular SMC Phenotype in Murine Atherosclerosis. Circ Res. 2021;129:992–1005. 10.1161/CIRCRESAHA.120.318479. 34615369 10.1161/CIRCRESAHA.120.318479 PMC8637935

[68] Cao G, Xuan X, Hu J, Zhang R, Jin H, Dong H. How vascular smooth muscle cell phenotype switching contributes to vascular disease. Cell Commun Signal. 2022;20:180. 10.1186/s12964-022-00993-2. 36411459 10.1186/s12964-022-00993-2 PMC9677683

[69] Wu H, Shen Y, Sivagurunathan S, Weber MS, Adam SA, Shin JH, et al. Vimentin intermediate filaments and filamentous actin form unexpected interpenetrating networks that redefine the cell cortex. Proc Natl Acad Sci U S A. 2022;119:e2115217119. 10.1073/pnas.2115217119. 35235449 10.1073/pnas.2115217119 PMC8915831

[70] Sarkar R, Meinberg EG, Stanley JC, Gordon D, Clinton Webb R. Nitric oxide reversibly inhibits the migration of cultured vascular smooth muscle cells. Circ Res. 1996;78:225–30. 10.1161/01.res.78.2.225. 8575065 10.1161/01.res.78.2.225

[71] Micsonai A, Wien F, Kernya L, Lee Y-H, Goto Y, Réfrégiers M, et al. Accurate secondary structure prediction and fold recognition for circular dichroism spectroscopy. Proc Natl Acad Sci U S A. 2015;112:E3095-103. 10.1073/pnas.1500851112. 26038575 10.1073/pnas.1500851112 PMC4475991

[72] Lin C, Hsu F-Y, Shih C-M, Cheng T-M, Wu AT, Cheng C-H, et al. Programmable MRI contrast switching for spatiotemporal mapping of thrombus maturation via enzyme-directed nanoprobe reconfiguration. Nano Convergence. 2025;12:53. 10.1186/s40580-025-00518-w. 41165914 10.1186/s40580-025-00518-w PMC12575922

