# Supplementary material for: Pathology-tailored nanotherapy via Galectin-3-targeted and triple-responsive nanoparticles enables multimodal therapy against aortic dissection
Source: J Nanobiotechnology. 2026 Jan 13;24:41. doi: 10.1186/s12951-025-04012-7 (PMC12809945; doi:10.1186/s12951-025-04012-7)
Supplement: Supplementary file 1 — Supplementary Material 1 [file 12951_2025_4012_MOESM2_ESM.docx]

Supporting Information (SI)

**Pathology-Tailored Nanotherapy via Galectin-3-Targeted and Triple-Responsive Nanoparticles Enables Multimodal Therapy against Aortic Dissection**

Chi Lin ^1, 2^, Min-Lang Tsai ^3^, Hsin-Yi Chao ^3^, Tsai-Mu Cheng ^4, 7^, Chun-Ming Shih ^5, 7^, Alexander T.H. Wu ^4, 7^, Chia-Hsiung Cheng ^1, 6, 7^, Chen Yuan Hsiao ^7, 8^, Hsin-Ying Lu ^7, 9, *^, Chun-Che Shih ^7, 8, 9, *^, Fwu-Long Mi ^1, 2, 6, 7, *^

^1^ Graduate Institute of Nanomedicine and Medical Engineering, College of Biomedical Engineering, Taipei Medical University, Taipei 11031, Taiwan

^2^ Department of Biochemistry and Molecular Cell Biology, School of Medicine, College of Medicine, Taipei Medical University, Taipei 11031, Taiwan

^3^ Department of Food Science, National Taiwan Ocean University, Keelung 20224, Taiwan, ROC

^4^ The PhD Program for Translational Medicine, College of Medical Science and Technology, Taipei Medical University, Taipei 11031, Taiwan

^5^ Division of Cardiology and Cardiovascular Research Center, Taipei Medical University Hospital, Taipei 11031, Taiwan

^6^ Graduate Institute of Medical Sciences, College of Medicine, Taipei Medical University, Taipei 11031, Taiwan

^7^ Taipei Heart Institute, Taipei Medical University, Taipei 11031, Taiwan

^8^ Department of Surgery, School of Medicine, College of Medicine, Taipei Medical University, Taipei 11031, Taiwan

^9^ Division of Cardiovascular Surgery, Department of Surgery, Wan Fang Hospital, Taipei Medical University, Taipei 11696, Taiwan

* Corresponding authors.

*E-mail addresses*: [hsinyinglu110@tmu.edu.tw](mailto:hsinyinglu110@tmu.edu.tw) (H.-Y. Lu), ccshih0603@tmu.edu.tw (C.-C. Shih), flmi530326@tmu.edu.tw (F.-L. Mi)

*Present address:* 250 Wuxing St., Taipei 11031, Taiwan.

**Figure S1. GPC analysis of MCP and molecular weight calibration.** The molecular weight distribution of MCP was determined by gel permeation chromatography (GPC), with dextran standards (1.3, 5, 10, 40, and 130 kDa) used for calibration. The peak molecular weight (Mp), number-average molecular weight (Mn), weight-average molecular weight (Mw), and polydispersity index (PDI) of MCP were calculated to be 11,001 Da, 12,620 Da, 16,890 Da, and 1.34, respectively.


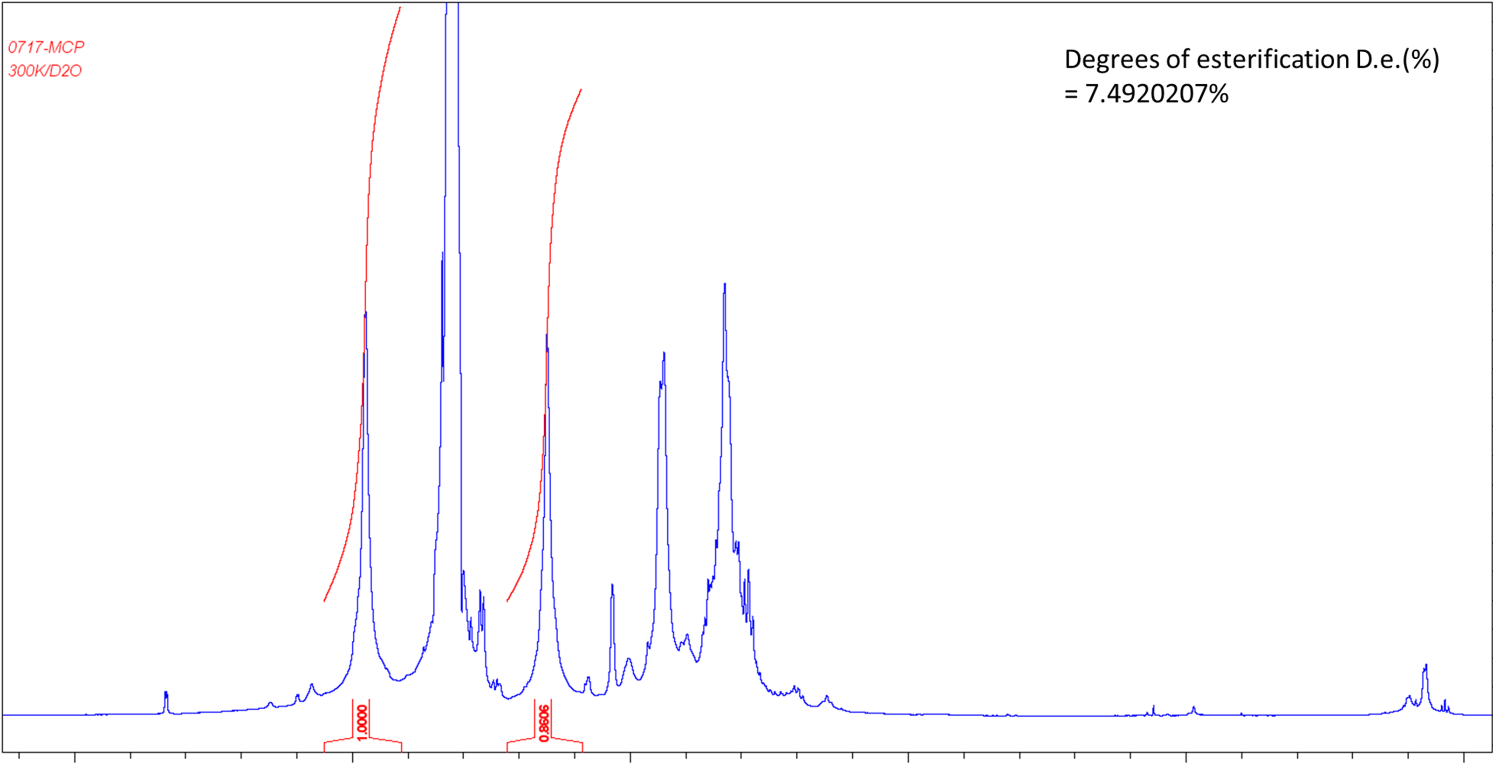


**Figure S2.** ^1^H NMR analysis of MCP for degree of esterification (D.e.) determination. The ^1^H NMR spectrum of MCP was recorded in D_2_O. The degree of esterification (D.e.) was calculated by integrating the proton signals at H1 (~5.0 ppm) and H4 (~4.3 ppm), using established integration ratios for galacturonic acid residues. The resulting D.e. was determined to be 7.49%.


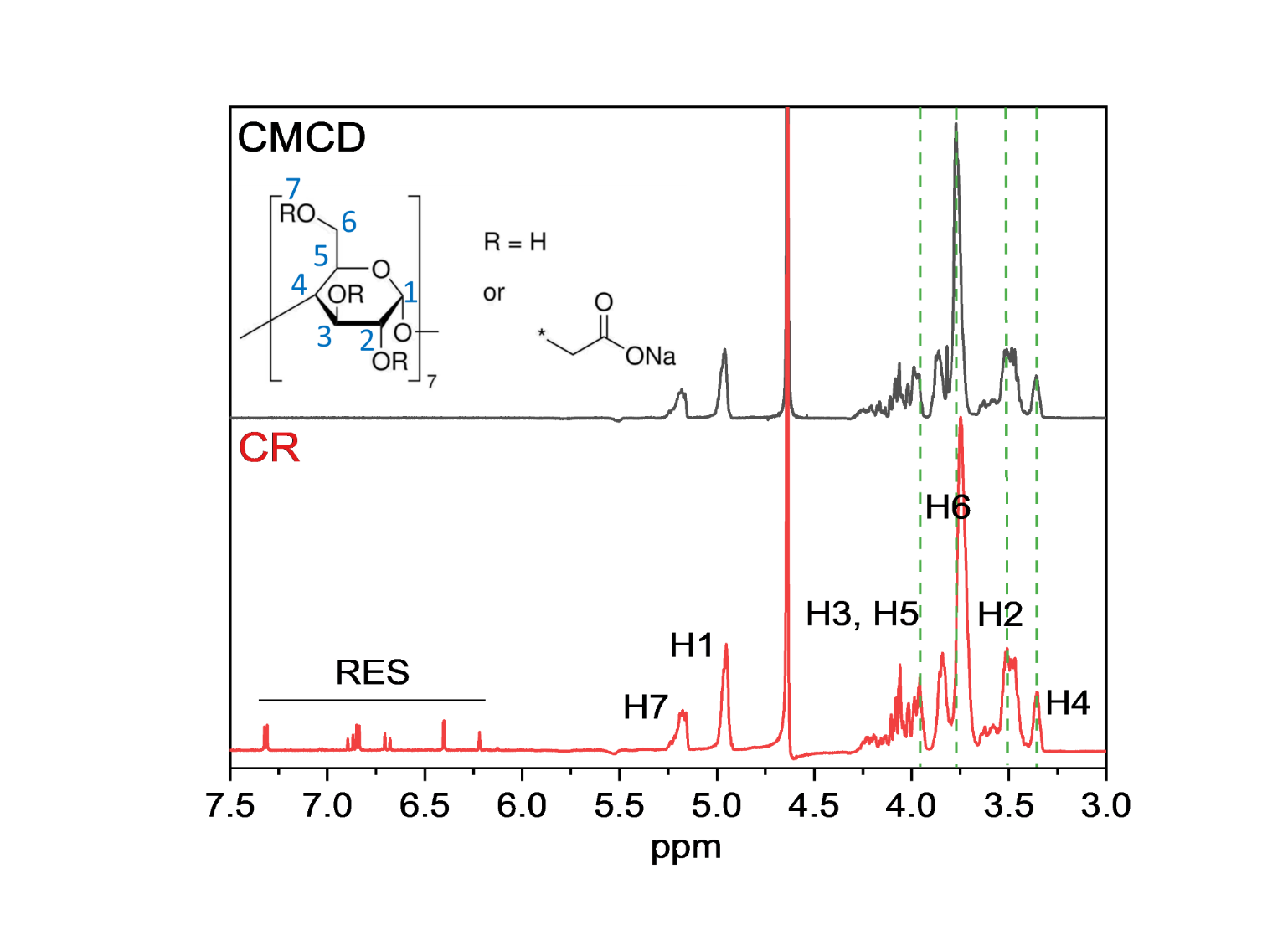


**Figure S3.** ^1^H NMR spectra of CMCD and CR in D_2_O, confirming the encapsulation of RES in CMCD. The characteristic aromatic proton signals of RES appear in the δ 6.2–7.2 ppm region, while the peaks corresponding to CMCD protons are assigned as H1–H7. Green dashed lines highlight the shifted proton signals after RES loading.


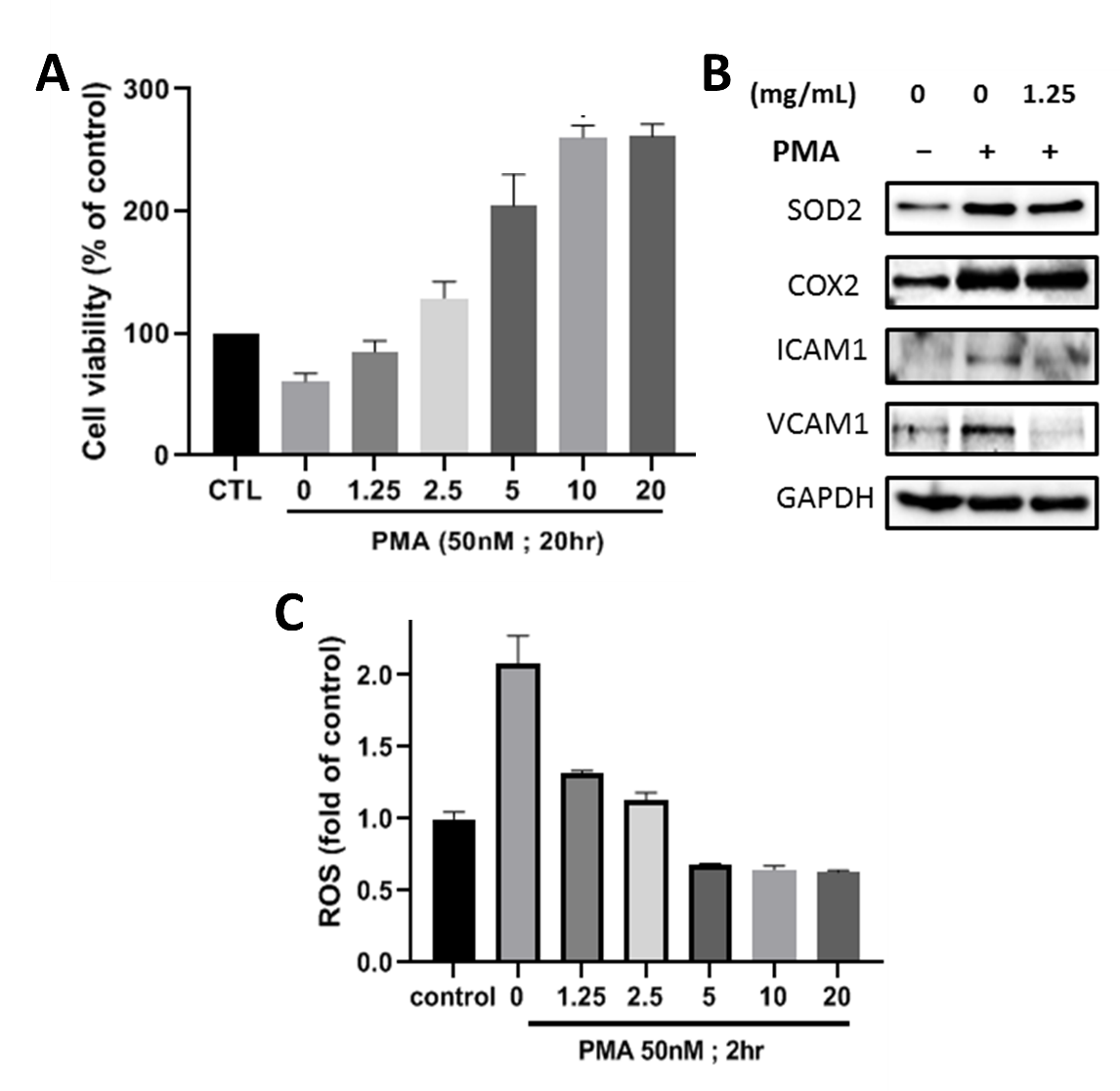


**Figure S4.** Protective effects of CR against PMA-induced inflammation and oxidative stress in vascular and immune cells. (A, B) HUVECs were treated with PMA (50 nM, 20 h) in the presence or absence of various concentrations of CR. (A) Cell viability and (B) expression of inflammation-related proteins (SOD2, COX2, ICAM1, VCAM1) were analyzed by CCK-8 assay and Western blot, respectively. GAPDH served as the loading control. (C) Intracellular ROS levels in PMA-stimulated THP-1-derived macrophages with or without CR treatment (50 nM PMA, 2 h).

**Figure S5.** FTIR spectrum of CP.

**
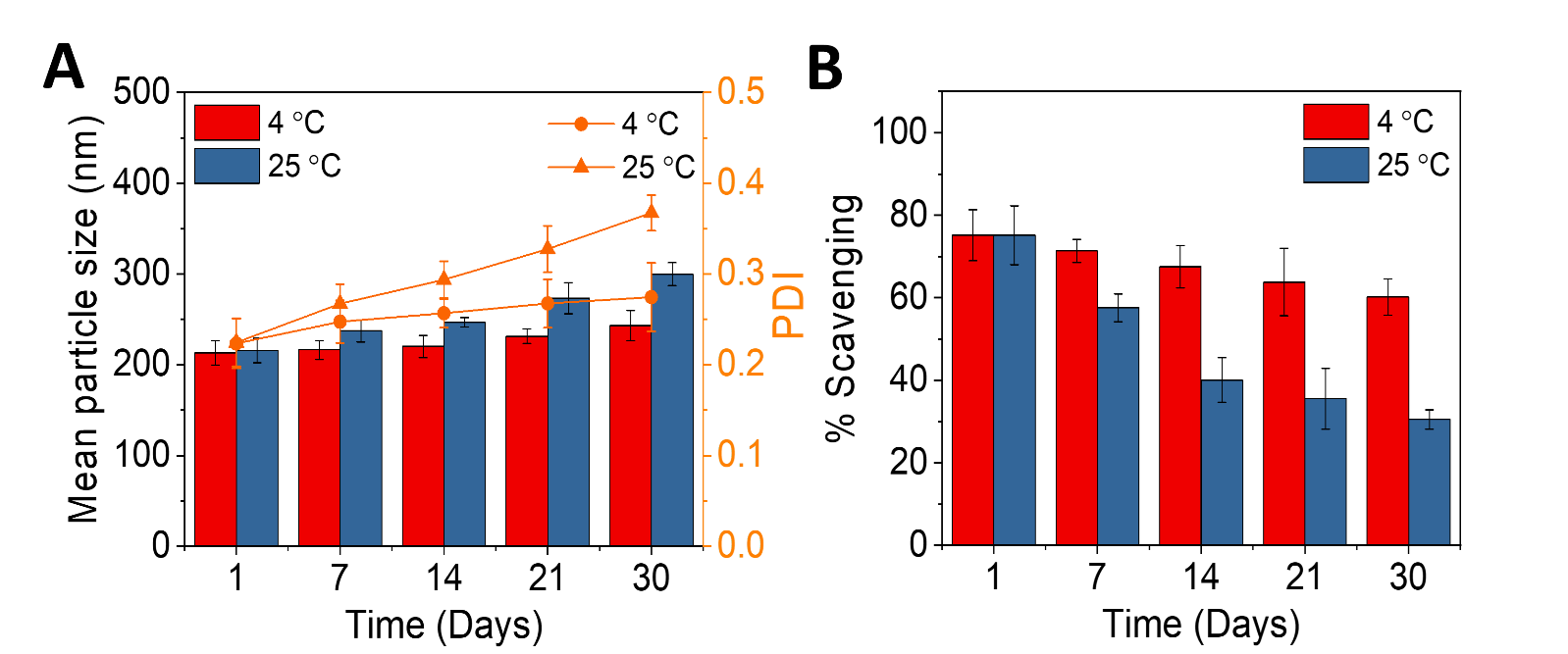
**

**Figure S6.** (A) Time-dependent changes in particle size and PDI of MPCR NPs stored in PBS at 4 °C or 25 °C for up to 30 days. (B) Corresponding antioxidant activity of MPCR NPs, measured by hydroxyl radical scavenging via ESR analysis (RES-equivalent concentration: 16 μM), after storage under the same conditions. MPCR NPs were freshly prepared and stored in sealed Eppendorf tubes, and samples were collected at designated time points for dynamic light scattering (DLS) and ESR assays. Data are presented as mean ± SD (*n* = 3).


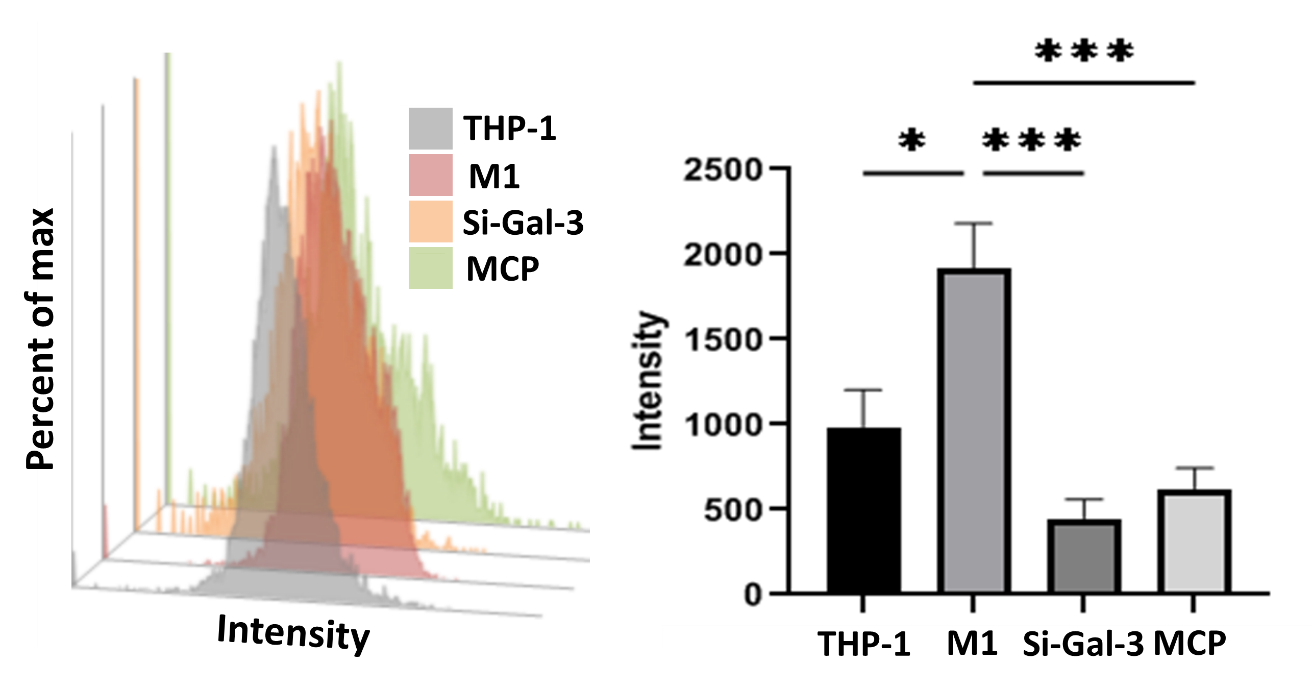


**Figure S7.** Flow cytometry analysis of MPCR NPs-FL uptake in THP-1-derived macrophages under different conditions. All groups were treated with MPCR NPs-FL (1 mg/mL, 1 h). Cells were pretreated with Gal-3 siRNA (si-Gal-3) or MCP (1 mg/mL, 1 h) prior to nanoparticle exposure. **p* < 0.05, ****p* < 0.001.

**
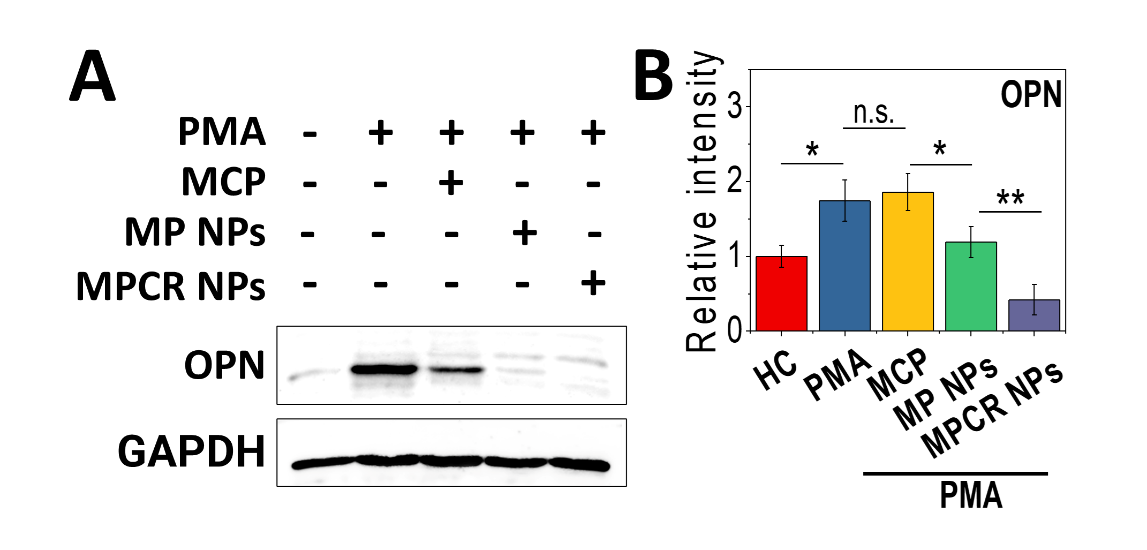
**

**Figure S8.** Western blot analysis of osteopontin (OPN) expression in VSMCs following PMA stimulation and treatment with MCP, MP, or MPCR NPs. GAPDH was used as the internal control. The bar graph shows densitometric quantification of OPN expression normalized to GAPDH (mean ± SD, *n* = 3). The corresponding uncropped Western blot images with molecular weight markers are shown in Fig. S12.

**Figure S9.** Pharmacokinetic profiles of resveratrol after intravenous injection of CR or MPCR NPs in AD mice. Mice bearing BAPN-induced AD were intravenously administered with CR or MPCR NPs at a RES-equivalent dose of 2.58 mg/kg. Blood samples were collected at predetermined time points (0, 0.25, 0.5, 1, 2, 3, 6, and 24 h), and plasma RES concentrations were determined using LC-MS/MS. Resveratrol-d₄ was used as an internal standard (100 ng/mL), and detection was performed in negative ion mode using multiple reaction monitoring (MRM) with transitions of m/z 227→185 for RES and m/z 231→189 for RES-d₄. Data are presented as mean ± SD (*n* = 4 per time point).


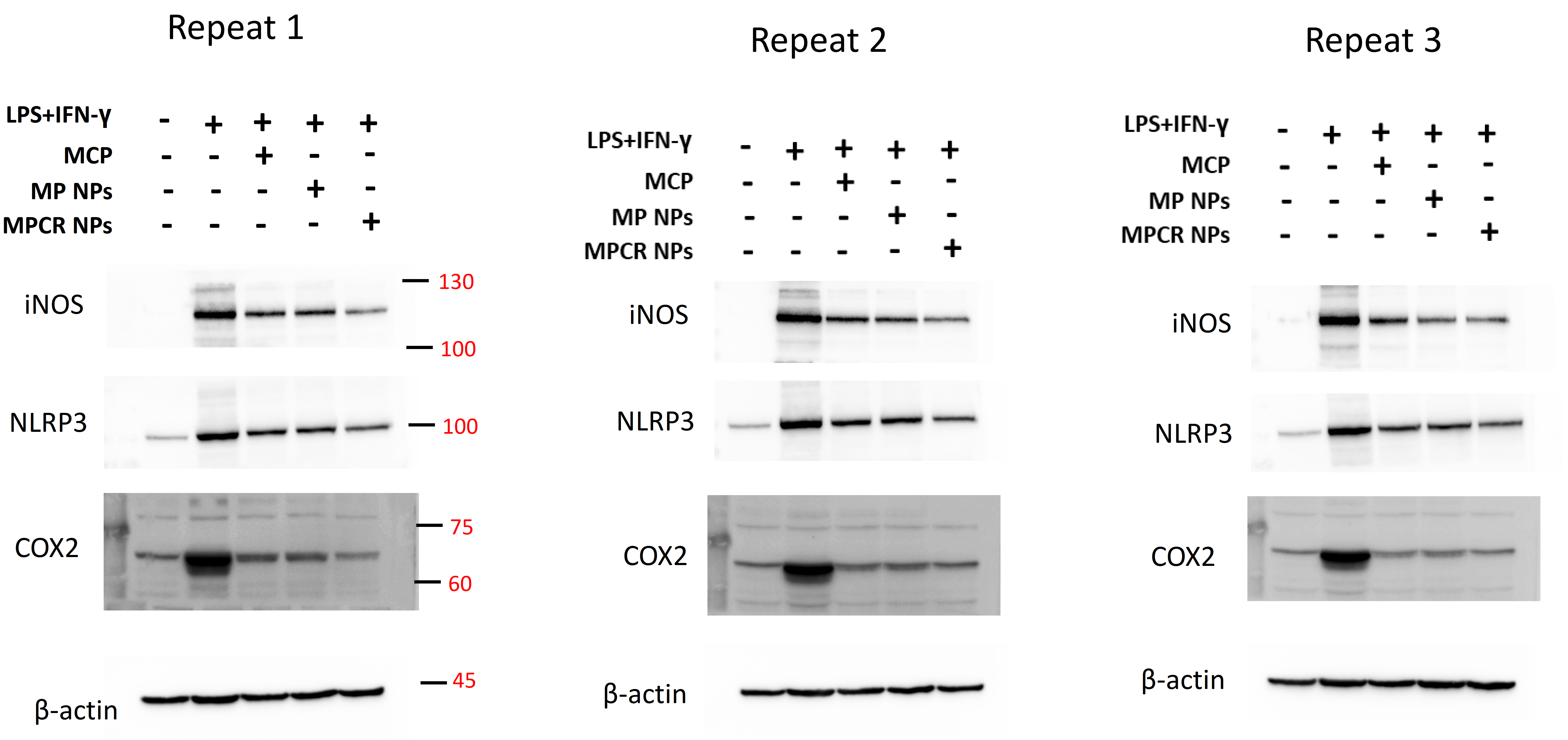


**Figure S10.** Full-length Western blot images corresponding to Fig. 7E, showing the expression of iNOS, NLRP3, and COX2 in RAW264.7 macrophages after treatment with LPS/IFN-γ, MCP, MP NPs, or MPCR NPs. Molecular weight markers are indicated on the right side of each blot. β-actin serves as the loading control.


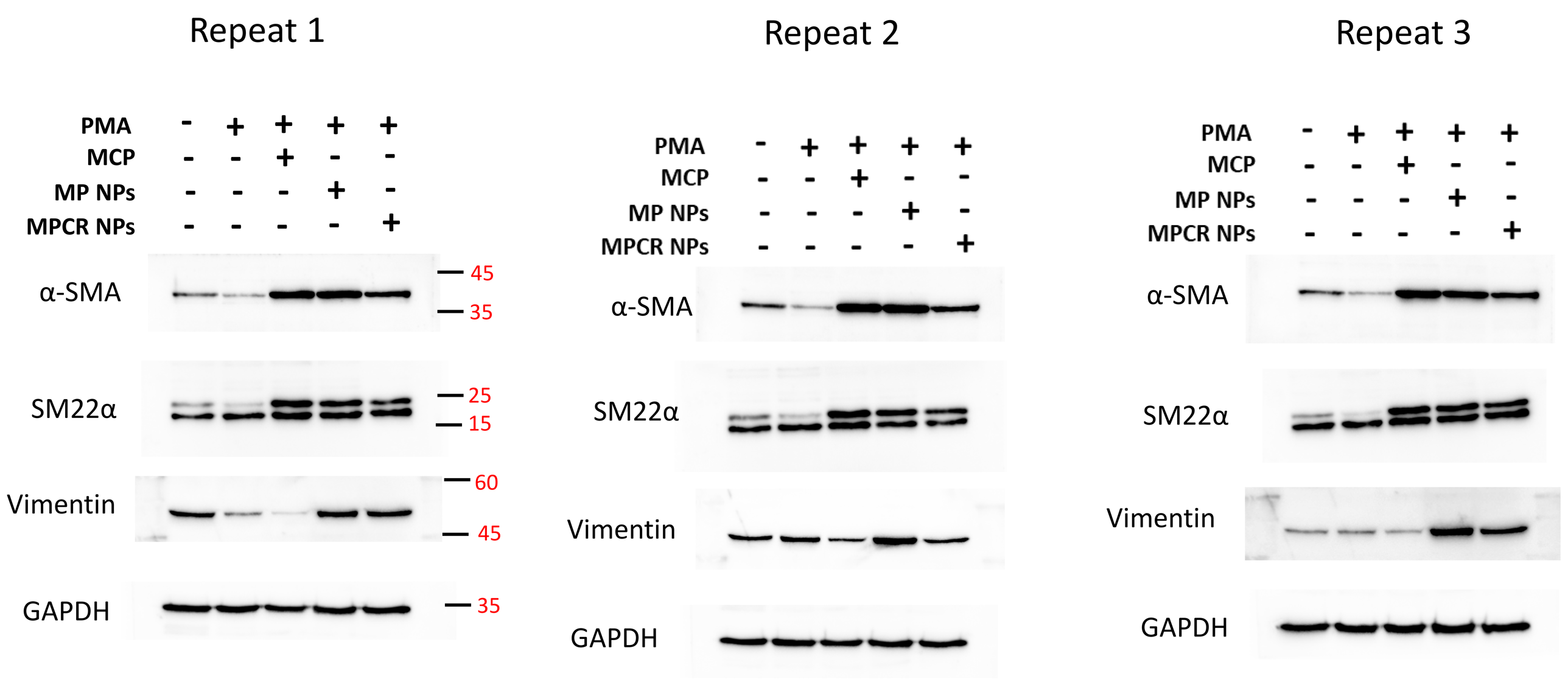


**Figure S11.** Full-length Western blot images corresponding to Fig. 7I, showing the expression of α-SMA, SM22α, and vimentin in primary aortic smooth muscle cells after treatment with PMA, MCP, MP NPs, or MPCR NPs. Molecular weight markers are indicated on the right side of each blot. GAPDH serves as the loading control.


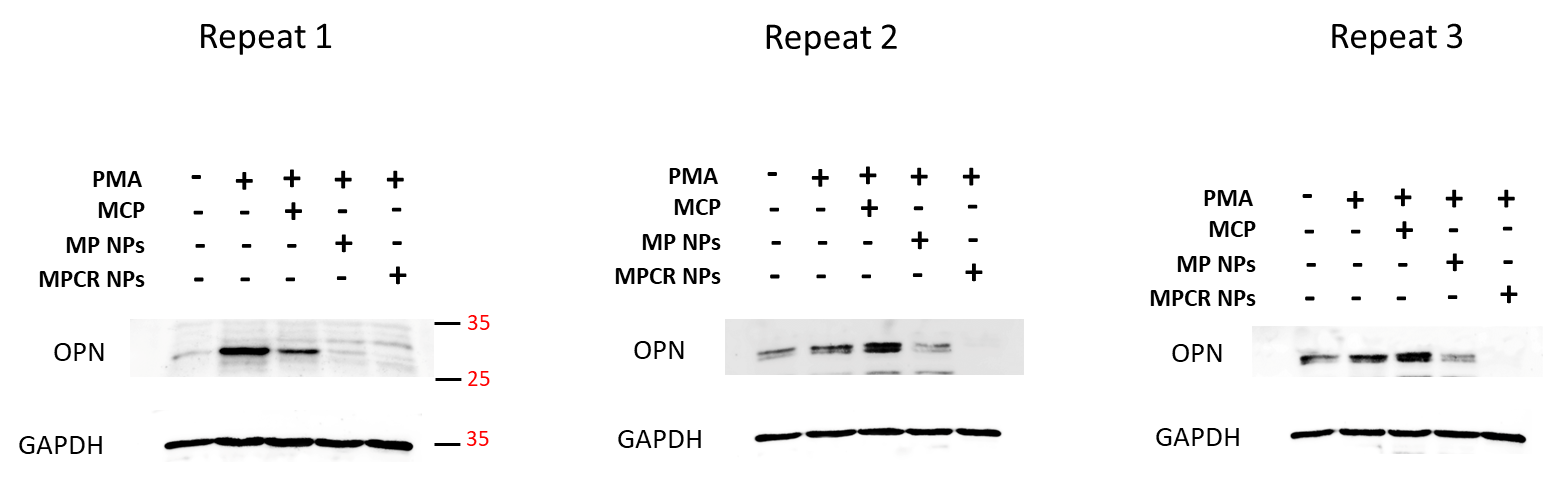


**Figure S12.** Full-length Western blot images corresponding to Fig. S8, showing the expression of OPN in primary aortic smooth muscle cells after treatment with PMA, MCP, MP NPs, or MPCR NPs. Molecular weight markers are indicated on the right side of each blot. GAPDH serves as the loading control.

**Table. S1** Physicochemical properties of various nanoparticle formulations, including hydrodynamic size, polydispersity index (PDI), and zeta potential.

|  | **Size (nm)** | **PDI** | **Zeta potential (mV)** |
| --- | --- | --- | --- |
| MP NPs | 240.6 ± 1.5 | 0.07 ± 0.008 | -12.8 ± 0.8 |
| MPC NPs | 215.8 ± 0.9 | 0.132 ± 0.016 | -13.6 ± 1.4 |
| MPCR NPs | 192.3 ± 2.2 | 0.152 ± 0.032 | -12.4 ± 4.3 |
| CDR/MP NPs | 232.2 ± 6.3 | 0.095 ± 0.092 | -10.6 ± 2.6 |
| PAA/MCP NPs-FL | 187.9 ± 3.5 | 0.082 ± 0.081 | -17.7 ± 3.4 |
| MPCR NPs-Cy7 | 195.6 ± 4.8 | 0.138 ± 0.025 | -14.6 ± 6.5 |
| MPCR NPs-FL | 191.1 ± 3.4 | 0.133 ± 0.042 | -13.2 ± 4.1 |

Values are presented as mean ± SD (n = 3).

**Table. S2** Percentage of secondary structures of Prt and the particles as determined by BestSel^TM^ from the respective CD spectra.

|  | α-helix | | β-sheet | | | | |  | β-turn | Random coil |
| --- | --- | --- | --- | --- | --- | --- | --- | --- | --- | --- |
| (%) | Helix-regular | Helix-distorted | Antiparallel-left twisted | Antiparallel-relaxed | Antiparallel-right twisted | Parallel | Total | |  |  |
| Prt | 0.0 | 2.6 | 5.3 | 12.1 | 14.8 | 0.0 | 32.2 | | 15.6 | 49.6 |
| MP NPs | 0.5 | 0.0 | 7.4 | 15.9 | 16.4 | 0.0 | 39.7 | | 15.1 | 44.7 |
| MPC NPs | 3.3 | 0.0 | 1.1 | 18.0 | 19.9 | 0.0 | 39 | | 20.7 | 37.1 |
| MPCR NPs | 0.0 | 0.0 | 0.4 | 11.3 | 13.7 | 0.0 | 25.4 | | 13.0 | 61.7 |
| Prt +CMCD | 0.0 | 0.9 | 0.0 | 12.9 | 15.3 | 0.0 | 28.2 | | 17.4 | 53.5 |

**Table. S3** Raw data used for generating Figure 1A, 1B, 1F, and 1G, including particle size and PDI values under varying MCPD, Prt, and CMCD ratios.

| MCP (mg) | Prt (mg) | CMCD (mg) | Size (nm) | PDI |
| --- | --- | --- | --- | --- |
| 0.5 | **1** | **-** | 933.1 ± 164.9 | 0.066 ± 0.053 |
| 0.5 | **0.83** | **-** | 794.3 ± 72.1 | 0.109 ± 0.051 |
| 0.5 | **0.66** | **-** | 772.1 ± 14.7 | 0.188 ± 0.03 |
| 0.5 | **0.5** | **-** | 667.7 ± 96.3 | 0.029 ± 0.002 |
| 0.66 | **1** | **-** | 870.8 ± 87.5 | 0.021 ± 0.069 |
| 0.66 | **0.83** | **-** | 965.3 ± 45.7 | 0.091 ± 0.009 |
| 0.66 | **0.66** | **-** | 607.4 ± 66.9 | 0.123 ± 0.046 |
| 0.66 | **0.5** | **-** | 242.7 ±1.9 | 0.061 ± 0.041 |
| 0.83 | **1** | **-** | 885.8 ± 140.2 | 0.054 ± 0.018 |
| 0.83 | **0.83** | **-** | 883.0 ± 8.1 | 0.128 ± 0.011 |
| 0.83 | **0.66** | **-** | 286.7 ± 0.2 | 0.089 ± 0.008 |
| 0.83 | **0.5** | **-** | 275.4 ± 1.9 | 0.035 ± 0.027 |
| 1 | **1** | **-** | 632.7 ± 9.5 | 0.222 ± 0.017 |
| 1 | **0.83** | **-** | 284.8 ± 1.2 | 0.098 ± 0.021 |
| 1 | **0.66** | **-** | 240.1 ± 0.9 | 0.074 ± 0.125 |
| 1 | **0.5** | **-** | 1145.2 ± 22.5 | 0.876 ± 0.065 |
| 1 | **1.33** | **3.33** | 845.9 ± 6.5 | 0.169 ± 0.018 |
| 1 | **1.33** | **6.66** | 853.3 ± 31.2 | 0.242 ± 0.038 |
| 1 | **1.33** | **10** | 707.3 ± 15.3 | 0.291 ± 0.081 |
| 1 | **1.33** | **13.33** | 536.6 ± 16.2 | 0.223 ± 0.064 |
| 1 | **1** | **3.33** | 387.5 ± 5.5 | 0.203 ± 0.031 |
| 1 | **1** | **6.66** | 372.1 ± 1.3 | 0.265 ± 0.024 |
| 1 | **1** | **10** | 279.6 ± 6.5 | 0.221 ± 0.026 |
| 1 | **1** | **13.33** | 269.1 ± 8.1 | 0.224 ± 0.001 |
| 1 | **0.66** | **3.33** | 225.4 ± 1.6 | 0.161 ± 0.009 |
| 1 | **0.66** | **6.66** | 215.6 ± 9.4 | 0.134 ± 0.046 |
| 1 | **0.66** | **10** | 217.2 ± 1.4 | 0.143 ± 0.028 |
| 1 | **0.66** | **13.33** | 228.2 ± 11.3 | 0.156 ± 0.038 |
| 1 | **0.5** | **3.33** | 255 ± 13.2 | 0.234 ± 0.038 |
| 1 | **0.5** | **6.66** | 274.5 ± 8.4 | 0.155 ± 0.018 |
| 1 | **0.5** | **10** | 263.8 ± 1.9 | 0.193± 0.028 |
| 1 | **0.5** | **13.33** | 272.3 ± 4.6 | 0.218 ± 0.013 |

Values are presented as mean ± SD (n = 3).
